# Limited synapse overproduction can speed development but sometimes with long-term energy and discrimination penalties

**DOI:** 10.1371/journal.pcbi.1005750

**Published:** 2017-09-22

**Authors:** Harang Ju, Costa M. Colbert, William B. Levy

**Affiliations:** 1 Informed Simplifications LLC., Earlysville, Virginia, United States of America; 2 Mad Street Den Inc., Fremont, California, United States of America; 3 Department of Neurosurgery, University of Virginia, Charlottesville, Virginia, United States of America; Université Paris Descartes, Centre National de la Recherche Scientifique, FRANCE

## Abstract

Neural circuit development requires that synapses be formed between appropriate neurons. In addition, for a hierarchical network, successful development involves a sequencing of developmental events. It has been suggested that one mechanism that helps speed up development of proper connections is an early overproduction of synapses. Using a computational model of synapse development, such as adaptive synaptogenesis, it is possible to study such overproduction and its role in speeding up development; it is also possible to study other outcomes of synapse overproduction that are seemingly new to the literature. With a fixed number of neurons, adaptive synaptogenesis can control the speed of synaptic development in two ways: by altering the rate constants of the adaptive processes or by altering the initial number of rapidly but non-selectively accrued synapses. Using either mechanism, the simulations reveal that synapse overproduction appears as an unavoidable concomitant of rapid adaptive synaptogenesis. However, the shortest development times, which always produces the greatest amount of synapse overproduction, reduce adult performance by three measures: energy use, discrimination error rates, and proportional neuron allocation. Thus, the results here lead to the hypothesis that the observed speed of neural network development represents a particular inter-generational compromise: quick development benefits parental fecundity while slow development benefits offspring fecundity.

## Author summary

The brain processes information by building and adjusting neural connections, and as an organism learns about the environment, it incorporates associations between features of the environment into these adjustable connections. Organisms, however, are not born with these connections; synapses must develop from a connectionless set of neurons. An important aspect of normal brain development is the timely development of the brain and of hierarchically arranged brain regions. In this paper, we show that an unsupervised algorithm, called adaptive synaptogenesis, builds neurons with stable connections and allows these neurons to appropriately discriminate patterns. Here, we show that larger rates of synaptogenesis and synaptic modification speed up development of stable connections. However, such a speed-up produces larger, steady-state energy-costs. We conjecture that these synaptic modification rates have evolved as a compromise via natural selection. In this regard, the evolution-theoretic idea of parent-offspring conflict arises as a developmental consideration; that is, time to stable connectivity must balance with subsequent neural performance. Importantly, we illustrate that the widely-observed phenomenon of synapse overproduction during development can be understood as a compromise between speedy and efficient development.

## Introduction

The phenomenon of net synapse overproduction in brain development is well-documented [1–4] but, as far as we can find, almost nothing definite is said about its purpose. Nevertheless, misregulation of this overproduction phenomenon is postulated to be a possible cause of autism [5–7]. The research reported here provides a functional perspective for two opposing goals in the regulation of synapse number. The first goal is that synapse overproduction speeds the time to develop a quasi-stable connectivity (from here on referred to as stable connectivity or just stability; see Methods for the need to qualify stable connectivity as quasi-stable rather than an absolute stability). The second goal is to avoid too many synapses per neuron for reasons of (i) energy efficiency of neuron-use [8, 9], (ii) per-category allocation of neuronal responses, and (iii) specificity of neuronal responses [10].

Empirical studies suggest that following overproduction, net synapse loss is integral to normal development [11, 12] including development of executive function [12] and memory performance [13]. Indeed, the results here support, in a quantitative fashion, Purves’s and Lichtman’s qualitative suggestion that “subsequent elimination of some innervation is a strategy well-suited to ensuring both the prompt innervation of all the cells in a target and ultimately, a quantitatively appropriate distribution of synapses among the target cells” [p. 135, 14; underscoring is our’s]. Moving from this qualitative statement toward the quantitative, in general, it might seem that faster development is better for the parents (particularly the dam), but there are other considerations; that is, the performance of neurons in the adult will also constrain development.

It seems that synapse overproduction appears in the computational literature only twice, and both times it is found in a figure without comment [10, 15]. The later study [10] shows that the synapse development paradigm of adaptive synaptogenesis [16, 17] can produce neuronal allocation in proportion to the input category frequency; this paradigm creates such a neuronal allocation in an unsupervised manner, without inhibition, and starting from a connectionless set of neurons. Here, we continue to investigate adaptive synaptogenesis. This model is inspired by BCM theory [18] and its functional control of activity (and we are not alone in considering functional controls over structural plasticity [19–21]). As used here, adaptive synaptogenesis incorporates (i) Hebbian synaptic modification, (ii) stochastic, postsynaptic activity-dependent synaptogenesis, and (iii) anti-Hebbian driven synapse elimination [16, 22]. Previous studies have shown that this paradigm can produce what appears to be a homeostasis in synapse number and neuron firing while encoding the statistics of the input environment into its connections [10, 23–26]. The paradigm can also reproduce the results of postnatal ocular dominance experiments [27], and it achieves compressive coding with small information losses [24].

The present study begins with variants of an earlier result concerning the use of adaptive synaptogenesis in discrimination learning and the resulting neuronal allocation that reflects input statistics [10]. (Indeed, all the quantified results reported here were qualitatively observed in [10] for A, B1, B2, and B3 datasets; see Fig 6 in [10].) In the context of allocation that is proportional to input statistics and low error rates as outcomes of development, we evaluate rates of synapse growth and modification (i.e., γ and ε). Higher rates of synapse growth and plasticity reduce the time to reach stable connectivity, but these higher rates raise the energy-costs of using the stabilized network and downgrade proportional neuronal allocation and specificity of neuronal responses. The same result is obtained by manipulating the initial number of synapses.

## Results

### Neurally relevant adaptive synaptogenesis

The adaptive synaptogenesis model was developed to explain the results of postnatal manipulations of sensory experiences, for example the effect of monocular deprivation on neuron allocation in V1 neocortex [27]. Our interest here is in extending this theory to earlier times in development. A standard result in experimental neuroscience is that neurons are allocated proportionately to the frequency of input patterns [28]. Studies of adaptive synaptogenesis produce this proportionality [10, 26, 27]. That is, there is greater neuronal representation for categories that occur more frequently. Before studying the simpler and more easily interpreted dataset A1, we remind the reader of the biological relevance of the theory by demonstrating biased allocation of orientation sensitive neurons via simulations of prenatal and postnatal development.

**Fig 1:**
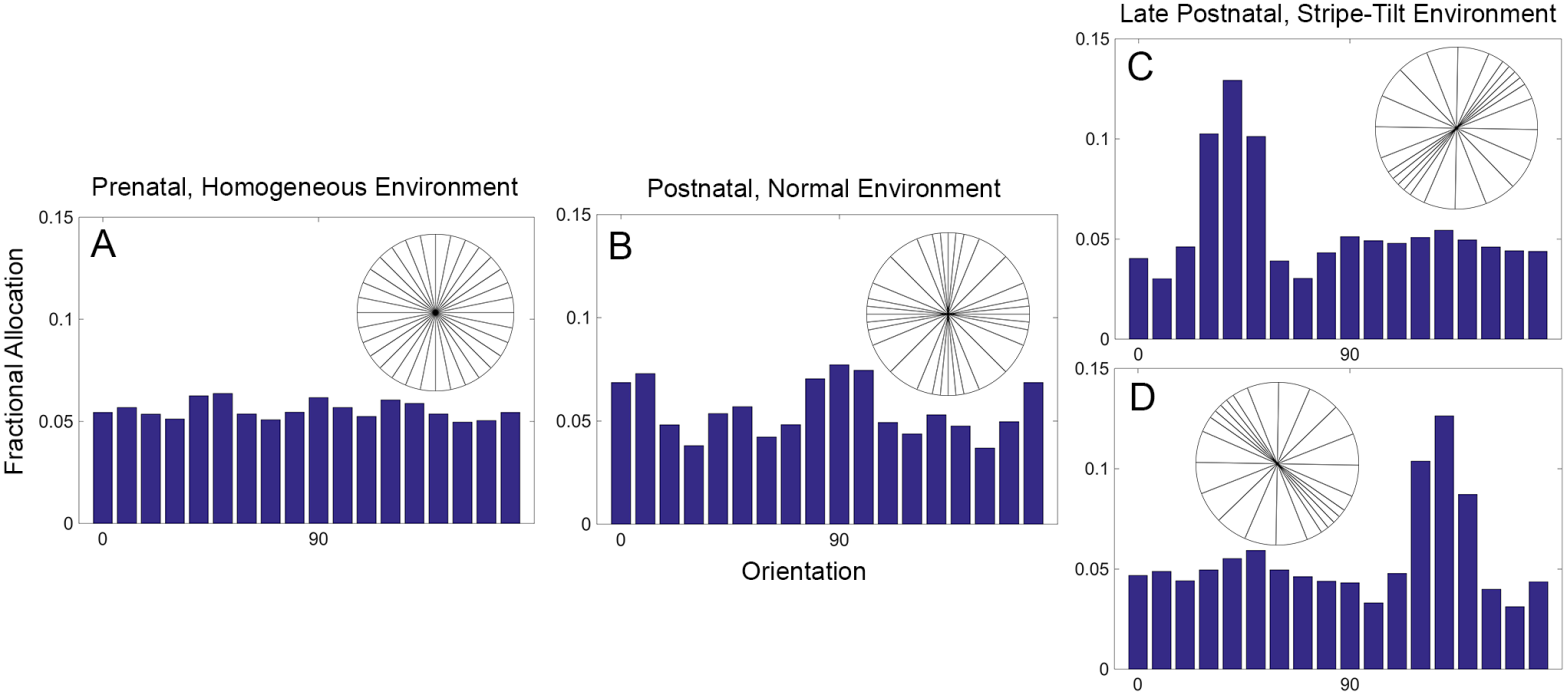
A sequence of input statistics across development biases allocation of orientation selective neurons. Adaptive synaptogenesis controls synaptic modification across three stages—one prenatal and two postnatal. In each stage, neuron allocation reflects the bias of the input statistics. Three input environments are presented sequentially, and synapses are allowed to stabilize in each environment. The allocation of neurons preferring each orientation reflects the probability of occurrence of the orientations. (A) Prenatal retinal waves with no orientation bias. (B) A natural, early postnatal environment with a slight bias for vertical and horizontal orientations. (C) and (D) show subsequent, experimentally manipulated stripe-tilt environments of 45 degrees and of 135 degrees, respectively. Each histogram bin represents an orientation ±5 degrees. The orientation circles are qualitative visualizations of the C datasets (Fig 9). Each histogram is based on 100 postsynaptic neurons.

The simulations make use of three environments: (i) a prenatal, retinal wave-generated environment with no bias in orientation (dataset C3), (ii) a normal postnatal environment with slight biases in the vertical and horizontal orientations (dataset C4), and (iii) a postnatal, experimentally manipulated, stripe-tilt environment with biases of 45 (dataset C1) and 135 (dataset C2) degrees. The three set of patterns were presented in sequence, one after another, with the algorithm running throughout; within each set, the patterns were randomly permuted. See Methods for more detail about the datasets and the references to the empirical motivations. All neurons begin with one connection. By the time the connections stabilize in the prenatal environment, a neuron has, on average, 9.4 connections, while in the normal postnatal and experimentally manipulated postnatal environments, a neuron has, on average, 11.1 and 12.1 connections. Stabilization in each environment produces different neuron allocations that reflect the orientation statistics of each environment (Fig 1). In the prenatal, uniform environment, neuron allocation is evenly spread across all orientations (Fig 1A). In the normal postnatal environment, more neurons are allocated to horizontal and vertical orientations (Fig 1B), and in the experimentally manipulated, stripe-tilt environment, orientations of 45 (Fig 1C) or 135 (Fig 1D) degrees receive the most neuronal allocation.

**Fig 2:**
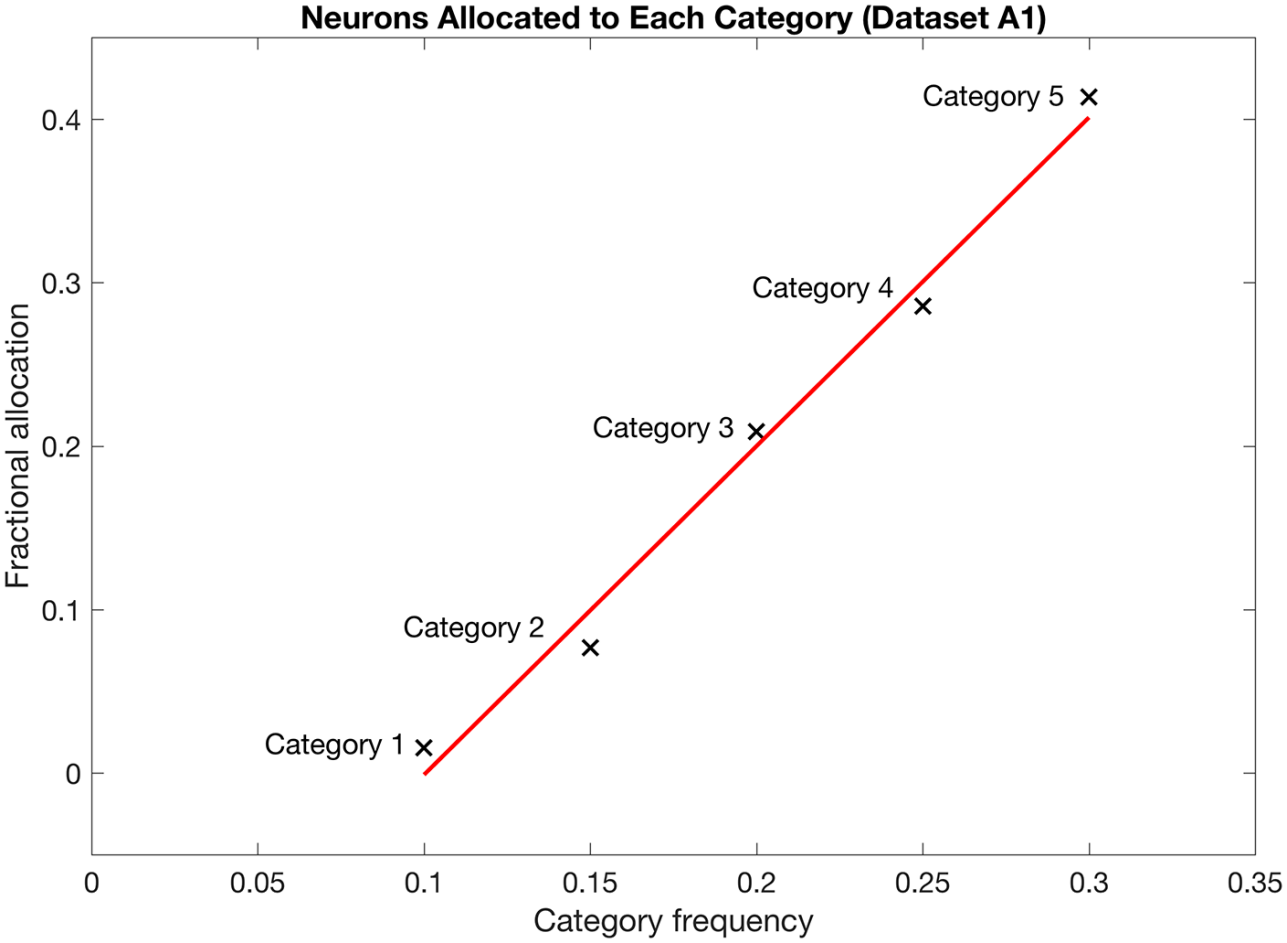
Neuron allocation suggests a linear relationship with category frequency. Each of 5 orthogonal prototypes defines an input category 1–5. Prototypes are never presented to the network. Input vectors are random perturbations of each prototype. See Dataset A1 in Methods (Fig 9). Once the connectivity of the simulation stabilizes, the allocation of postsynaptic neurons’ firings suggests a linear relationship to each category’s relative frequency. The simulation uses 100 postsynaptic neurons with γ and ε of 0.001 and a receptivity threshold shutoff of 0.1.

Because there is a certain complexity to the orientation datasets due to the overlaps between adjacent orientations [10], we turn to a simpler, non-overlapping environment with five categories of patterns, each with a different frequency of presentation to the network. The results of this section establish a baseline of neuronal allocations required of later results. Within dataset A1, neuronal allocation is a linear function of category frequency (the fraction of presentations of patterns generated by a certain prototype). Fig 2 illustrates this linear relationship. The five orthogonal categories with fractional frequencies of 0.1, 0.15, 0.2, 0.25, and 0.3 receive fractional neuron allocations of 0.01, 0.08, 0.21, 0.29, and 0.41, respectively. The linear regression line of Fig 2 has a slope of 2.0 and a r^2^ value of 0.99.

### Higher rates of synaptogenesis (γ) and synaptic modification (ε) speed stabilization of connectivity

**Fig 3:**
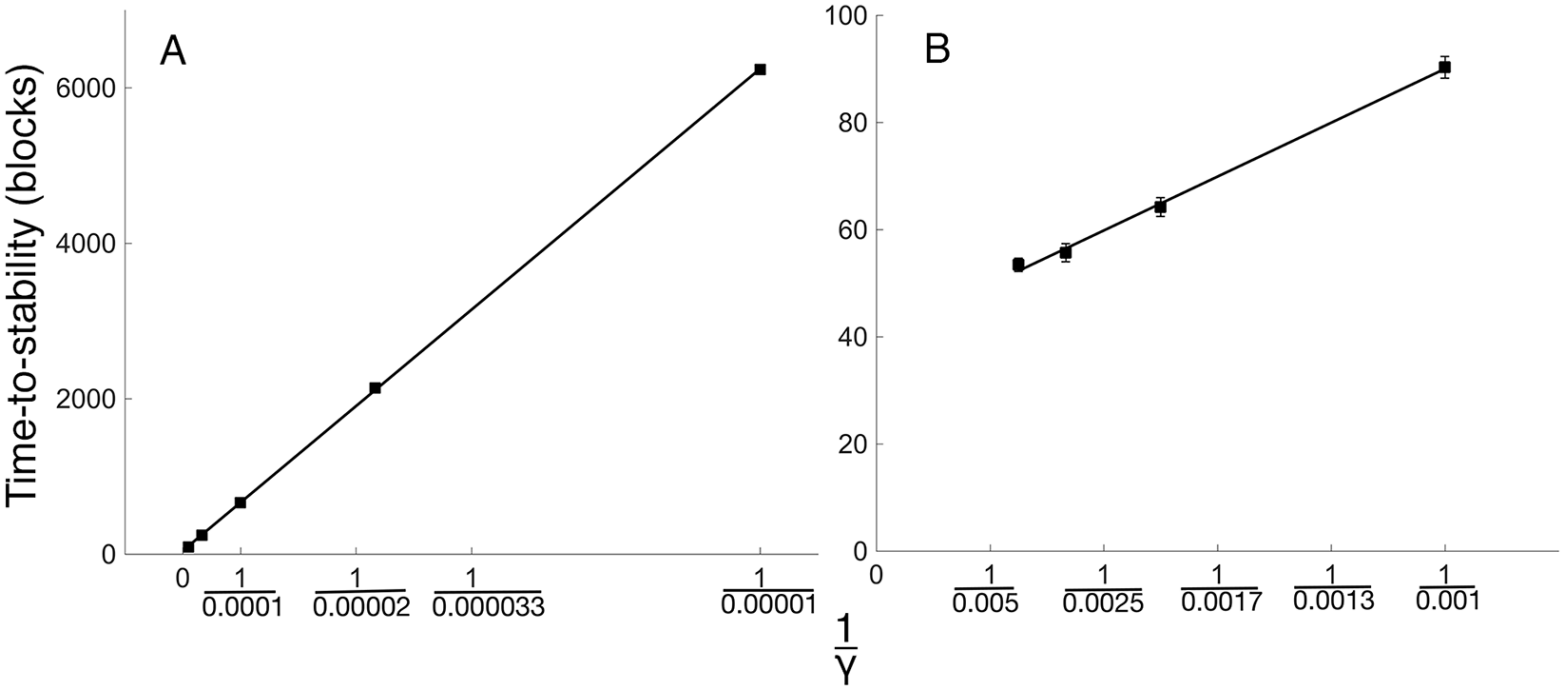
Time-to-stability is linearly related to the inverse of the probability of synapse formation. Increasing γ speeds development, and time-to-stabilize is proportional to the inverse of γ. The relation spans a considerable range, here plotted for both smaller (A) and larger (B) values of γ. (A) The γ values are 0.00001, 0.00003, 0.00001, 0.00003, and 0.001. (B) plots the same relationship on an expanded scale with γ values of 0.001, 0.002, 0.003, and 0.004. At each γ setting, an ε setting that produces the fastest development is used (See Methods and S1 Appendix). The error bars, if not visible, are smaller than the plotted points. Each data point is a simulation of 1,000 postsynaptic neurons.

Raising the rates of synaptogenesis (γ) reduces the time to reach stable connectivity (Fig 3). In conjunction with γ, the rate of synaptic modification (ε) also determines the time to reach stability. Thus, in Fig 3, the value of ε is the empirically observed optimum for each value of γ. See Methods and S1 Appendix Fig 3 for details of the empirical optimization of ε values.

Fig 3 plots time to stable connectivity as a function of the inverse of γ. There are two plots in Fig 3 because of the 500-fold range of γ values simulated; i.e., Fig 3A shows the results for the smaller γ values, and Fig 3B shows the results for the larger γ values. The two figures are in good agreement; the regression lines of Fig 3A and 3B have slopes of 0.06 and 0.05, respectively, and the same r^2^ values of 0.99. Using much larger γ values (e.g. 0.1) eliminates the linear relationship shown in Fig 2.

### Faster stabilization correlates with costlier neurons

**Fig 4:**
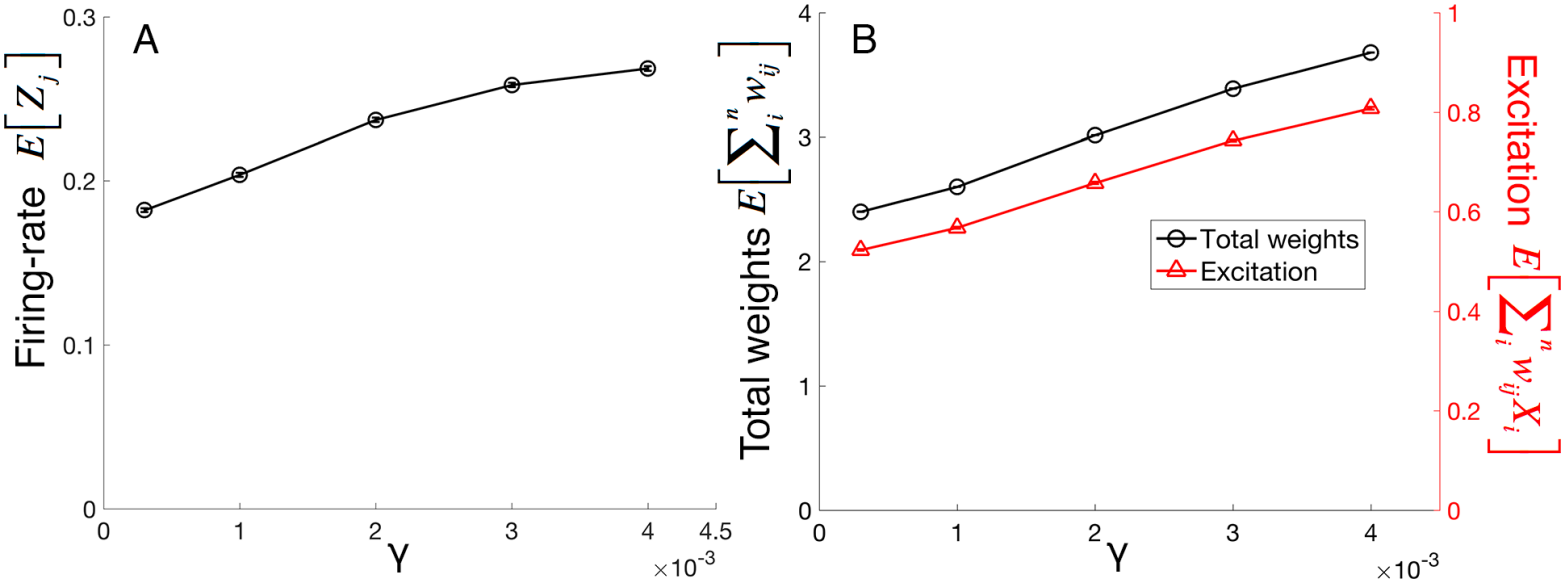
Increasing γ produces energetically costlier neurons. At stability, neurons that develop under larger γ’s use more energy in these ways. (A) Larger γ produces neurons with greater firing-rates. Communication costs rise with more action potentials because an action potential’s energy-cost is about 100 times the leak energy-costs over the same time interval. (B) Larger γ produces neurons with greater total synaptic weight and greater average excitation. The computational costs arise from maintenance of synapses, proportional to the total synaptic weight, and the average use of the synapses, proportional to average synaptic activation. The error bars, if not visible, are smaller than the plotted points. Each data point is a simulation of 1,000 postsynaptic neurons. Without knowing the constants c_ap_, c_leak,axo_, c_exc_, and c_leak,syn_, the y-axes do not afford comparisons across dependent variables.

Faster development mediated either by large γ is problematic when energy-costs are considered since faster development (i.e., shortest time-to-stability) correlates with greater energy-costs (Fig 4). Note that in Fig 4, there are there different axes for three different costs, each of which is defined in Methods.

**Fig 5:**
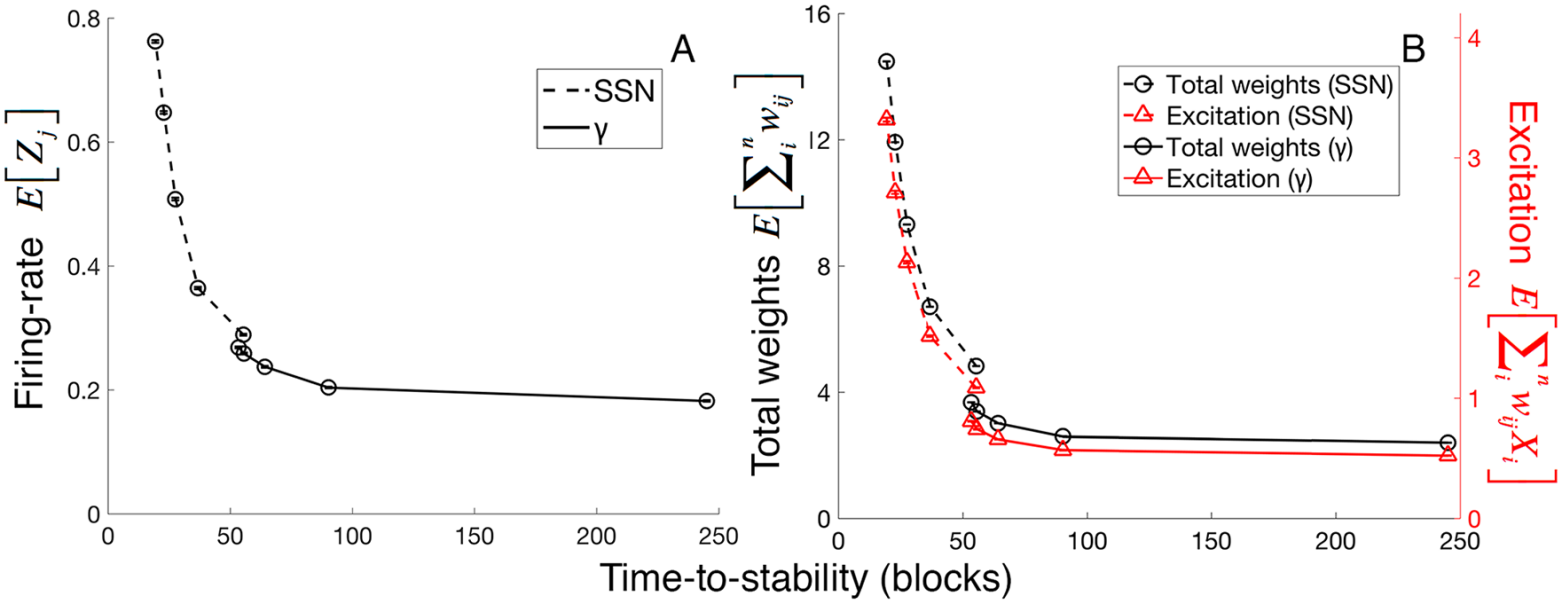
Faster development correlates with more energetically expensive neurons. (A) Neurons with shorter time-to-stability have more frequent action potentials and thus greater communication costs on average. (B) Neurons with shorter time-to-stability have greater total synaptic weights and greater average synaptic activation. The plotted points on the dashed lines come from simulations manipulating the starting synapse number (SSN). The values of SSN for the points from right to left are 50, 100, 150, 200, and 250 out of 1,000 total possible synapses. These simulations use fixed values of γ and ε at 0.001. The plotted points on the continuous lines come from simulations manipulating γ. The values of γ for the points from right to left are 0.0003, 0.001, 0.002, 0.003, and 0.004. Optimal values of ε are used for each γ (See Methods and S1 Appendix). The error bars, if not visible, are smaller than the plotted points. Each data point is a simulation of 1,000 neurons. Without knowing the constants *c*_*ap*_, *c*_*leak*,*axo*_, *c*_*exc*_, and *c*_*leak*,*syn*_, the y-axes do not afford comparisons across dependent variables.

A postsynaptic neuron’s energy-costs arise from its computation and communication costs. Computational costs of the neuron *j* arise from (i) maintenance of its total number of synapses *m*_*j*_, and (ii) the average use of these synapses, a value proportional to *E[Y*_*j*_*]*. Communication costs arise from time-proportional leak and action potentials, where action potentials require about 100 times the energy-cost of leak per unit time [8]. Thus, communication costs increases with firing-rate. See Methods for further explanations of the measurements of energy-cost. Fig 4 plots each of these relative costs as a function of γ; the greater the value of γ, the greater the firing-rates, the greater the average internal excitations, *E[Y*_*j*_*]*, and the greater the synapse numbers. Combining these results (Fig 4) with those of the previous section (Fig 3) reveals an inverse relationship between time-to-stability and energy-costs of neuron-use (Fig 5).

### Faster stabilization correlates with less selective neurons and loss of proportional neuronal allocation

As firing-rate increases for a neuron, its error-rate also increases. An error-rate is defined as the fraction of the time a neuron fires to a pattern belonging to a non-preferred category (see Methods). The largest error-rate is six times the smallest. The error-rates are steadily increasing with number of synapses, and this increase is what causes the gradual loss of specificity. The error-rates are 0.0097, 0.0135, 0.0279, 0.0435, 0.0616 for the sequence of average number of synapses: 15, 16.8, 20.27, 23.45, and 25.8.

With larger γ, the neuronal allocation that was a linear function of category probability (see Fig 2) is lost. For example, when γ is 0.1, categories one through five have fractional neuronal allocations of 0.02, 0.06, 0.10, 0.41, and 0.40, respectively.

### Increasing starting synapse number (SSN) while holding γ and ε constant

In addition to increasing γ, initializing a simulation with more, random connections also reduces time-to-stability. Initializing neurons with as many as 100 (out of 1,000 possible) randomly chosen synapses, as opposed to an initialization of just one synapse, speeds development while still achieving the appropriate neuron allocation (as illustrated in Fig 2). In this particular case, the time-to-stability is cut by 60%. Fixing values of γ and ε at 0.001, SSNs of 1, 50, 100, and 150 lead to an average time-to-stability of 90.9, 55.4, 36.8, and 27.6 blocks, respectively.

The relationship between time-to-stability and energy-costs is consistent for both manipulations of γ (continuous lines in Fig 5) and SSN (dashed lines in Fig 5). A simulation with higher SSN has even shorter time-to-stability and even larger communication and computational costs than simulations with larger values of γ. At an SSN of 100 and γ and ε values of 0.001, a neuron reaches stability in 36.8 blocks on average, whereas a neuron at an SSN of 1, a γ value of 0.001, and an ε value of 0.0038 reaches stability in 56.7 blocks on average. However, the energy-costs of the neuron developed with 100-SSN are much higher than the neuron developed at 1-SSN. The percent increase for each cost from 1-SSN are 180% for firing-rate, 267% for excitation, and 258% for total weight per neuron, a substantial increase in each measure.

Though simulations with SSN values greater than 100 produce even faster development, the discrimination performance of neurons in these simulations is severely prone to error. For example, with an SSN of 150, the average firing-rate is 0.5 when stability is achieved; that is, a neuron at 150 SSN fires 50% of the time. Each category in these simulations is, however, presented 30% of the time or less. Thus, even when one assumes that such neuron fires 100% of the time to the most frequent category with its 30% appearance rate, it must be firing 20% of the time to other categories. If the neuron prefers a category that is presented less than 30% of the time, the error rate can only be worse. Thus, SSN-based overproduction also diminishes the specificity of neuron firing.

### Synapse overproduction correlates with fast stabilization

Overproduction of synapses is a well-known phenomenon of development and, at first glance, seems energetically wasteful. This section points out why limited overproduction may exist.

**Fig 6:**
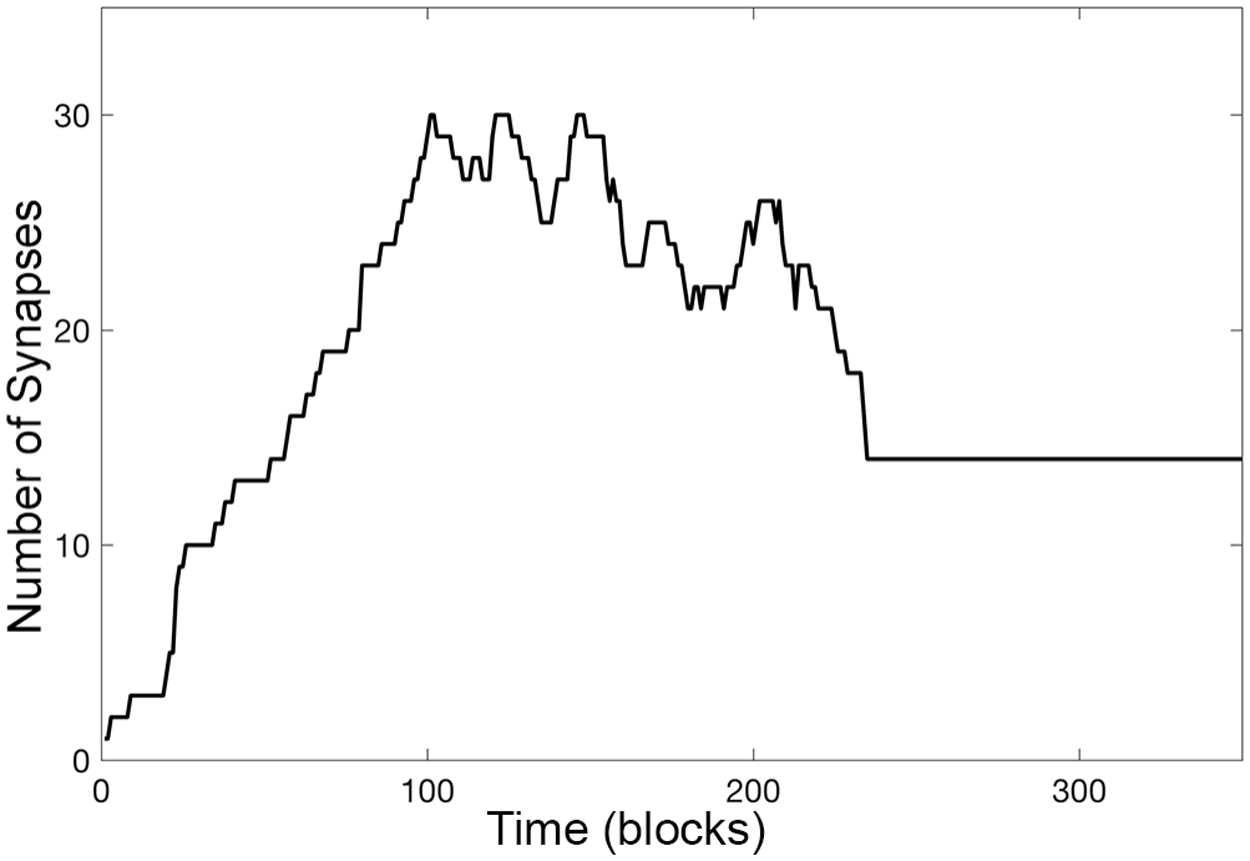
A neuron overproduces and subsequently sheds synapses. An individual example of the overproduction phenomenon. The neuron develops in a simulation with a γ value of 0.0003 and an ε value of 0.0015 and is representative of the amount of overproduction for neurons with these settings. It obtains a maximum total synapse number of 30 at block 101, but eventually stabilizes with 14 synapses by block 235. The simulation continues for 1,088 more blocks with connections remaining unchanged. Note that the example neuron shown here (Fig 7B) is chosen from the 1,000 neurons used to produce the single data point where γ equals 0.0003.

For the adaptive synaptogenesis paradigm, synapse overproduction characterizes fast development; the greater the overproduction, the faster the development. By definition, an overproduction occurs when a neuron gains more synapses than are present at stabilization; clearly at some point during development, there must be more shedding than synaptogenesis. Fig 6 illustrates a single neuron example. Starting with one connection, this neuron eventually stabilizes at 14 synapses. However, it first achieves 14 synapses at block 52 and continues gaining synapses; it eventually reaches a maximum synapse number of 30 at block 101. Following this maximum, there is a period of oscillations, but on average, there is a decrement in synapse number until around block 200; at this point in development, shedding proceeds with very little synaptogenesis leading to a stable synapse number of 14 at block 235. (The simulation continues for 1,088 more blocks with synaptic connections remaining unchanged.)

**Fig 7:**
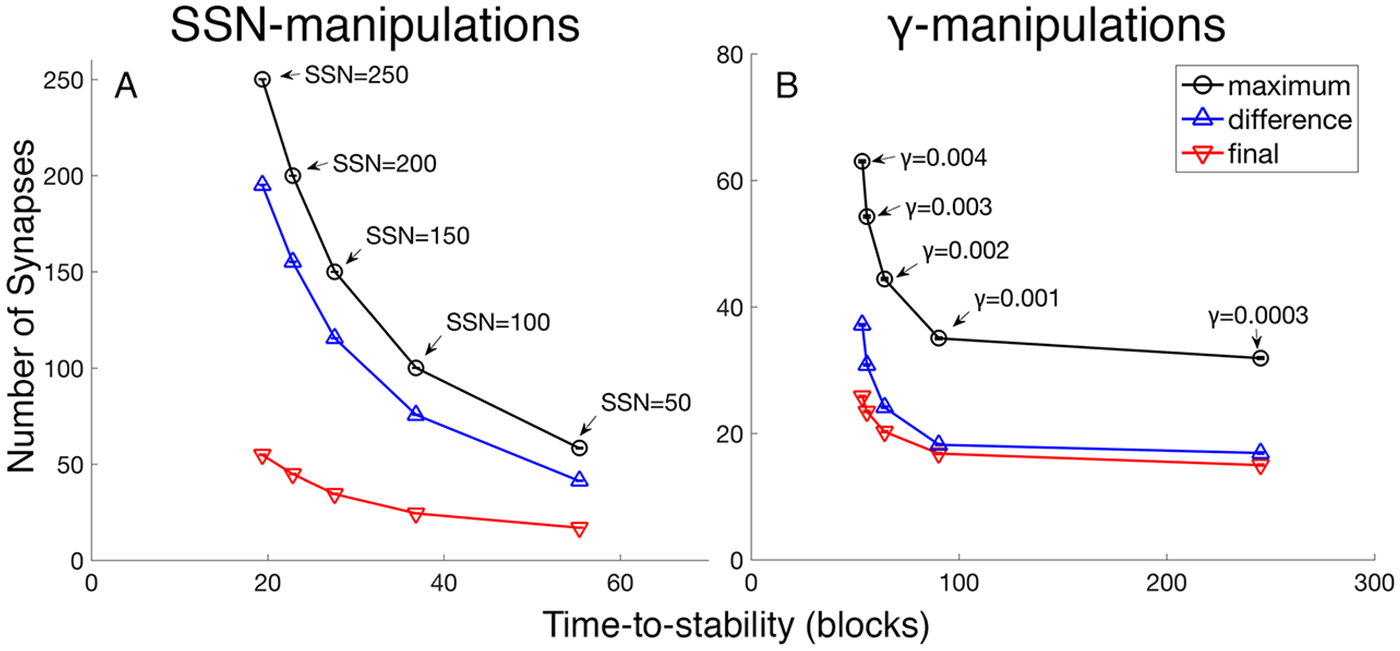
A transient overproduction in the number of synapses is more prominent in fast developing neurons. Faster developing neurons have larger overproductions in synapse number as well as more synapses at stability. The overproduction is calculated as the difference between each average maximum (black, circle) and the corresponding average final number of synapses per neuron (red, upside-down triangle). (A) As SSN is increased, overproduction becomes more prominent. For simulations manipulating SSN, the values of γ and ε are fixed at 0.001 for all points, and the values of SSN for the points from left to right are 50, 100, 150, 200, and 250 out of 1,000 total possible synapses. (B) As γ is increased, neurons converge faster, and overproduction (red, circle) is more prominent. These simulations manipulating γ use values of ε that are fastest for the given value of γ (See Methods for how such values of ε are found), and the values of γ for the points from left to right are 0.0003, 0.001, 0.002, 0.003, and 0.004. Each point is a mean of 1,000 postsynaptic neurons.

The size of this overproduction effect increases in concert with the speeding of development; this correlation is true for both γ or SSN manipulations (Fig 7A). Fig 7 presents direct comparison of the average maximum (red/square line) value to its corresponding average stable value (black/circle line). At a γ value of 0.001 and SSN of 1, for example, on average, the maximum synapse number is 35 accompanied by an average stable value of 17; by calculation then, the average overproduction is 18. For this same SSN of 1 and the largest acceptable γ of 0.004, the average overproduction is 37.7. For a γ value of 0.001 and SSN of 100, the average overproduction is 75.6 synapses.

## Discussion

The main contributions of this work are (i) a model of synapse overproduction with explicitly delineated control mechanisms and (ii) bringing attention to a fitness hypothesis that constrains neural development: specifically, duration of neural development as a phenotype (an idea implicit in the mentioned quote of Purves and Lichtman concerning rapid development).

For the adaptive synaptogenesis paradigm presented here, this study elucidates three microscopic, arguably evolvable, parameters that control a neuron’s rate of afferent synaptic development. In this regard, increasing SSN (i.e., quick synapse formation in the absence of associative modification) and increasing the value of γ (the rate of synaptogenesis) are just the starting points. As noted in the Methods section, for speediest development, ε must be matched to both the input statistics and γ’s value. Regardless, development that is too speedy leads to certain undesirable concomitants. Under the developmental model studied here, there is an inverse relationship between a neuron’s time to form stable connections in a statistically stationary environment and the energy-costs of the steady-state synapses. In addition, proportional neuronal allocation is lost when development is too quick. These effects of increasing γ generalize to a more complex input environment; see S2 Appendix.

### Why is speed of development important?

Earlier studies of neuron optimization have concentrated on the energy-cost of information processing [29–32] and the energy-cost of communication [8]. These sensible perspectives are not broad enough, at least for us, given the added context of synaptic development. With this additional context, a lifespan perspective (prenatal to adult) motivates our questions about adaptive synaptogenesis [33–38]. The demonstrations here illustrate our working hypothesis that natural selection creates a balance between speed of connectivity development and the later costs of using the relatively stabilized connectivity.

Inspired by this line of thinking, the report here notes the existence of an antagonistic interaction between two phenotypes that are both determinates of fitness in the modern Darwinian sense (i.e., lifespan and inter-generational theories of fitness; see above references and S3 Appendix for more about our research philosophy including our perspective on optimizations [39]). The two phenotypes are (i) the duration of neural development, a time period requiring the reproductive efforts of time and energy by the parents as they intensively nurture their developing offspring [37, 38], and (ii) the implied adult behavioral performance and its adult costs associated with these same offspring. In the case of (i), the costs include the energy-expense to the parents of providing for the entire organism of each of the offspring (not just neural energy-costs) and, additionally, the opportunity-loss incurred by the delay in starting a new brood; both of these costs increase with longer prenatal development-time and increase with the duration from birth to weaning. In the case of (ii), the costs take the form of poorly apportioned neuronal allocation, assuming the importance of well-apportioned neural codes (cf. Figs 1 and 2; see also discussion in 10), and the energy associated with activating synapses (cf. Figs 4 and 5). As far as we are aware, the antagonism between time-to-develop and later adult quality-of-performance has not yet influenced quantitative control of neural development. The parametric study here, which hypothesizes such an antagonism, offers a rational explanation for limited synapse overproduction, at least for those who believe in a lifespan perspective for defining fitness.

### Synapse overproduction

Net synapse elimination characterizes certain phases of cortical development. (Net synapse elimination refers to the net loss in the number of synapses, as opposed to synapse elimination that is part of synapse turnover, a process that may not result in a change of synaptic totals.) Both non-human studies [1, 40–44] and human studies [45–47] show net synapse elimination occurs in various parts of the brain at various times in development. For example, a human brain undergoes synapse growth prenatally and for a few months after birth. Net synapse elimination then begins postnatally and accelerates at onset of puberty [48]. Some studies observe synapse elimination even in young adults, both in humans [49] and rats [50].

A primary hypothesis of the present research is that synapse overproduction reflects the speeding of development in order to reach an approximate steady-state of connectivity. However, our model is not the only synaptogenesis model that finds the existence of overproduction. Another postsynaptic activity-dependent paradigm also creates overproduction. Although observed without comment, this other model [15] produces a similar correlation between overproduction and time-to-stability as elucidated here.

In focusing on synapse overproduction, the research here first points out that two fundamental parameters control overproduction—the {ε, γ} pair and SSN. Second, it points out that too much overproduction, which does indeed continue to increase the speed of development, can cause later problems. From this perspective, observing net synapse elimination in normal development is observing the result of a compromise, and from our perspective, an evolvable one.

The two manipulations, (i) {ε, γ} and (ii) SSN, are natural controls of synaptic development; moreover, there is the requirement that {ε, γ} must be appropriately matched for best results, yet another part of the overall optimization problem studied here (see Methods). The similarity of the results using the two distinct manipulations, i.e. (i) and (ii), is notable. Both control mechanisms lead to the same problems when applied in an excessive manner. Specifically, too much synapse overproduction leads to excessive adult energy-costs because (i) acquiring many weak synapses at random implies that there will be more positively correlated input lines for whatever category wins the competition for a neuron’s allocation (Fig 7) and because (ii) more stabilized synapses consume more energy. Moreover, accompanying the largest overproduction are two more problems: first, neuron allocation unduly favors the high probability categories while in the second case, a neuron is less likely to be category specific in its responses when it stabilizes with too many synapses.

Importantly, the SSN observations point to the generality of the results. When SSN is large, the parameters controlling synaptogenesis—γ, ρ and $\bar{Z_{j}}$, become irrelevant to the formation of synapses. The mechanism of synaptogenesis is irrelevant because with large SSN, only the bidirectional Hebbian modifications and the shedding rule come into play; synaptogenesis never occurs.

The biological meaning of SSN is worth a comment since it might not be obvious. Our interpretation of SSN as modeled here is a very early phase of very fast synaptogenesis (i.e., a large γ) in the absence of any Hebbian or anti-Hebbian modification, i.e., ε = 0. Of course, γ has several physical possibilities, including a growth factor-dependent rate of probing axonal growth or a value reflecting the availability of postsynaptic territory on which to form new synapses.

### Alternative models and future extensions

With small alteration, the approach here can be melded with BCM theory. For example, one can use the combination of SSN, the BCM synaptic modification equation, and the shedding principle. However, expanding upon adaptive synaptogenesis with the Δw equation used here holds our interest.

In terms of the generality of adaptive synaptogenesis, the reader is cautioned that the specific theory presented here will benefit from, or even require, certain modifications. First, not all synapses of forebrain cortical systems are expected to use synapse overproduction as studied here. Second, different classes of synapses will have different sets of modification rules, and in such cases, the ideas here will have to be modified for successful application. For example, inhibitory and monoamine synapses require different considerations. Similarly, it can be questioned whether this theory applies to (a) excitatory feedback synapses (perhaps half the synapses of cortex) or to (b) lifelong associatively dynamic encoders (e.g., neurons of the hippocampal system [51] or the short-term memory system of dorsal medial prefrontal cortex). That is, the neurons and synapses of such regions present a particular challenge; because the neurons of these regions are constantly changing their identity (i.e., the stimulus to which they best respond), they require a more nuanced definition of quasi-stability compared to cortical region V1.

One particular result, the excessive firing-rate—that is an observed concomitant of overly rapid synaptogenesis—will be easy to control in a more comprehensive model. For example, and still using adaptive processes, mechanisms such as a local, adaptive threshold-adjustment or some forms of inhibition, with their own set of synaptic modification rules, can be used to force firing-rates to some prescribed level. As one form of such inhibition, lateral inhibition might succeed in improving discriminability of categorical representations. Of course, adding lateral inhibition in the form that exists in neocortex only seems sensible when the input has an appropriate topological property (which can be added to adaptive synaptogenesis, as found in our own preliminary results; see also Ooyen and van Pelt [15]). But still topology, and thus, lateral inhibition are vexing issues when considering clusters defined in high dimension as opposed to the rather simple computations involved in early sensory processing where spatial clustering is inherent (e.g., retinotopy, tonotopy, etc.). Again considering either inhibition or local threshold modification, these enhancements are less than cure-alls: neither of these mechanisms will affect the synapse overgrowth and the associated adult energy costs; secondly, it is not at all obvious that these mechanisms can recover proportional neuronal allocation when there is the overabundance of synapses as occurs along with very fast development; and then there is the problem of piling too many adaptive processes on top of each other while preserving the existence of quasi-stability. Only in regard to this last objection is there a simple solution, a developmental critical period, which times-out synaptogenesis, i.e., modulates γ to zero.

When expanding a model, one must always check to see that the base functions are not lost. In this regard, it seems sensible to alter the present model when incorporating mechanisms that can by themselves control firing-rates. That is, to incorporate inhibition or local threshold modification forces us to backtrack a little and use a synaptogenesis control-mechanism closer to our original approach [16, 23, 27]. Specifically, if one uses other mechanisms to control firing rate E[*Z*_*j*_], then adaptive synaptogenesis should be curtailed based on the moving average of internal excitation *Y*_*j*_, not based on the moving average of firing-rate *Z*_*j*_. Thus, on top of a *Y*_*j*_ shutoff-control of synaptogenesis, there can be an additional adaptive process without the stabilization problem mentioned above. Another advantage of controlling synaptogenesis with the average value of net excitation is that this approach optimizes information-rate and the associated synaptic energy-use (see [10] Theory section). Then separately, other adaptive algorithms can control firing rate, which itself figures into the large energy-cost of long-distance communication. Preliminary simulations of *Y*_*j*_–based synaptogenesis indicate that the major relationships shown here still hold; that is, small overproduction of synapses is good for fast development, but even faster development results in energetically costlier neurons in the sense of acquiring additional excitatory synapses without any benefit to function.

A final enhancement of the current model, and one that we consider rather urgent, is a control mechanism for the rate constants ε and γ. The success of the current model and many other computational models of neural network development depend on the ergodic mixing characteristics of the input. This assumption seems sensible prenatally and early postnatally. However, the stimuli of the postnatal environment, particularly in the context of high-level, neocortical processing, are far from ergodic. Thus, upgrading the model to handle the absence of input ergodicity is, for us, an active area of research.

Relative to brain function, the assumptions underlying the goals of any empirically observed developmental process (e.g., synaptogenesis) is bound to be contentious. However, we submit that the final arbiter will include the perspective of evolution through natural selection rather than merely the perspective of neuroscience alone. Such a broadened approach can benefit the priorities of empirical neuroscience. For example, there may be a relatively small number of genes that control SSN or γ, making these variables a tempting target for empirical research.

## Methods

### Neurons

This study uses adaptively constructed, feedforward networks of McCulloch-Pitts neurons [52]. For the synapses that exist, an input vector *x(t)* at time-step *t* produces internal excitation *y*_*j*_*(t)* of neuron *j* with weights *w*_*ij*_*(t)*.

$$y_{j}\left(t\right)\;=\;\sum_{i}x_{i}\left(t\right)\;\cdot \;w_{ij}\left(t\right)$$

where *x*_*i*_(*k*) ∈ {0,1} and *w*_*ij*_(*k*) > 0. If the internal excitation is greater than a predetermined threshold *θ*, neuron *j* produces an output firing. That is,

$$z_{j}\left(t\right)\;=\;\left\{1\;if\;y_{j}\left(t\right)\;>\;\theta ,\;and\;0\;oth\text{e}rwise\right\}$$

No interaction exists between the outputs of these neurons (i.e. there is no feedback or lateral inhibition), and each neuron develops its connections independently of all other neurons.

### Adaptive synaptogenesis

There are three parts to adaptive synaptogenesis: synaptogenesis, associative synaptic modification, and synaptic shedding.

Synaptogenesis, when allowed to occur, is a random Bernoulli process with parameter γ and is governed by *r*_*j*_*(k)*, a neuron *j*’s receptivity for synaptogenesis at the conclusion of block *k*. A block consists of a complete set of randomized inputs (see Timescales). This receptivity depends on a constant γ and the moving average of neuron *j*’s firing-rate. The moving average $\bar{z_{j}}\left(k\right)$ is updated after each block *k* of input presentations.

$$\bar{z_{j}}\left(k\right)\;=\;\bar{z_{j}}\left(k\;-\;1\right)\;\cdot \;\alpha \;+\;\left(1/n_{t}\right)\sum_{t=1}^{n_{t}}z_{j}\left(k,\;t\right)\;\cdot \;\left(1\;-\;\alpha \right)$$

where α = 0.25, *n*_*t*_ is the total number of exemplars in a block. Neurons that fire above rate ρ will not add new synapses. When a new synapse is formed, its initial weight is set to 0.1, an arbitrarily chosen value greater than the shed weight. Synaptogenesis is allowed after each presentation of a block of input patterns.

$$r_{j}\left(\text{k}\right)\;=\;\{\gamma \;if\;\bar{z_{j}}\left(k\right)\;<\;\rho ,\;and\;0\;otherwise\}$$

where *ρ* = 0.1

Associative synaptic modification [10, 17] alters weights and captures correlations in the inputs. Given a binary input vector *x(t)* such that *x*_*i*_(*t*) ∈ {0,1} and rate constant ε, where *t* advances one with each time-step.

$$w_{ij}\left(t\;+\;1\right)\;=\;w_{ij}\left(t\right)\;+\;\varepsilon \;\cdot \;\left[x_{i}\left(t\right)\;-\;E\left[x_{i}\right]\;-\;w_{ij}\left(t\right)\right]\;\cdot \;y_{j}\left(t\right)$$

Synaptic shedding [25] occurs when the weight of a synapse is below 0.01. Because a *de novo* synapse is given a weight of 0.1; it is not immediately subject to shedding. No negative weights exist.

$$w_{ij}\left(k\right)\;=\;\{0\;if\;w_{ij}\left(k\right)\;<\;0.01,\;and\;w_{ij}\left(k\right)\;otherwise\}$$

### Timescales

In the adaptive synaptogenesis model, there are three timescales (from shorter to longer duration): 1) the presentation of an input pattern (i.e. one time-step *t*), to which a neuron fires or not; 2) the synaptic modification of existing synapses as determined by the rate constant ε; and 3) synaptogenesis and shedding. Synaptic modification occurs after each presentation of an input pattern, and synaptogenesis and shedding are allowed to occur after presentation of a block *k* of input patterns. The rate constants, ε and γ, determine the exact timescales of synaptic modification, shedding, and synaptogenesis. In one block, all input vectors are presented to the network in a randomized order.

An input block consists of a sequence of randomly chosen prototypes that are each randomly perturbed. The random prototype selection is well-mixed. For each postsynaptic neuron, input blocks are presented until no synapses are gained or lost for 200 blocks, at which point a neuron’s synapses are assumed stable. At this time, the weights have achieved their values predicted by the adaptive synaptogenesis theory [10]. See the section Input datasets for the block sizes for each input dataset.

### Parameters

For the purpose of the study, values for parameters ε and γ are manipulated (see Adaptive Synaptogenesis in Methods). The parameter values are listed in Table 1.

**Table 1:** Network parameters.

Parameters	Values
ε, rate constant for synaptic modification	manipulated
γ, rate constant for synaptogenesis	manipulated
α, rate constant for $\bar{z_{j}}$	0.25
ρ, threshold for receptivity, *r*_*j*_*(k)*	0.1
θ, threshold for neuron firing, $\bar{z_{j}}\left(k\right)$	1.0
Number of initial connections for a neuron	manipulated
Initial synaptic weights	0.1
Shedding value	≤ 0.01

#### Setting the variables of ρ, θ, and α

A great deal of pilot simulations preceded the research presented here; this pilot work determined the settings for firing threshold, the moving-average time constant the determines the moving average firing-rate, and the cutoff value that halts synaptogenesis onto *j*. In arriving at the settings and in general, we entertain the following assumptions: (i) cortical neurons have many excitatory synapses which leads to desirable statistical computations, employing the benefits of the law of large numbers and the central limit theorem; (ii) although a biological neuron has many inputs, it only sparsely samples the full input space (that is, the set of all the presynaptic axons to the neuron’s brain region); (iii) firing rates are low, on average much lower than 50%, and (iv) the moving-averages that encode statistics are good approximations of the corresponding expectations because they are matched to the ergodicity and mixing of the input exemplars.

These assumptions lead to some very crude approximations and demand compromises, particularly in terms of (i) and (ii). The input dimension of a sensory cortical region is huge, well beyond anything we can repetitively simulate and analyze. Likewise, the number of synapses per neuron is also large, in the thousands, while the sampled subspace, as a fraction of the total number of input axons, is inevitably very small; its value is less than a fraction of one percent in visual cortex. With these thoughts in mind and with tractable input dimensions ranging from 100 to 400, we settled on the desirable number of synapses per neuron in the range of 20–50. By achieving around 20 synapses per postsynaptic neuron, some of the benefits of the central limit theorem just start to be seen. It is the Central Limit Theorem and the setting of threshold that allows these neurons to demonstrate relatively high specificity for the very noisy input datasets. Given the input datasets used here, θ and ρ are set to achieve this range of final synapse-number per neuron. Increasing ρ increases the number of synapses and decreases the specificity of neuronal responses.

The value of α should be matched to the ergodicity of the inputs. The goodness of this approximation is determined by how well mixed the input samples are and by the variation of the input firing vectors. It is assumed that, in utero and even for a brief part of the postnatal period in altricial species, input vector statistics are predictable enough for evolution to match the moving-average rate-constants that it imparts to neurons matching the random variation of the input environment. Later postnatal times, when the input world might not be so well mixed, modulatory factors will be needed to dynamically adjust rate constants. This problem of dynamic adjustment is left for future research.

Given assumptions (i)-(iv) and parameters set to obey these assumptions, we believe our synapse overproduction results will be robust even across certain variants of adaptive synaptogenesis.

### Quantification of dependent variables

#### Time-to-stability

The time-to-stability is measured as the number of blocks required both for (i) the number of synapses and for (ii) the weights of synapses to converge to a statistical steady-state. When no synapses are gained or lost for 200 presentations of input blocks, neurons are considered to have converged, fulfilling requirement (i). Based on an earlier study [10], 200 blocks of unchanging synapse number were enough for the weights to stabilize on average, fulfilling requirement (ii) and also to reach the values predicted by the adaptive synaptogenesis theory [10]. It should be noted that such a the steady-state is possible because the random inputs are forced to be well-mixed. In fact, for a fully randomized input, this is a quasi-steady-state due to the possibility of a very improbable occurrence of a long, poorly mixed sequence.

#### Neuron allocation index

The neuronal allocation index per category is the fraction of total neuron firings to a single category. For example, suppose that ten neurons fire a total of 100 times to an input set with three categories: category one accounts for 20 of these firings, category two accounts for 30 of these firings, and category three accounts for 50 of these firings. Then, categories one, two, and three will respectively have neuron allocation indices of 0.2, 0.3, and 0.5. To determine the neuron allocation index, a testing set consisting of a novel set of 100 input blocks is presented without modifying weights.

#### Firing-rate

The firing-rate is calculated by averaging the firings of a neuron that is presented with a testing set of input patterns without synaptic modification occurring during these presentations.

#### Energy-cost

The energy-cost formula has four terms: for two synaptic, computational costs, ($c_{leak}E\left[\sum_{i=1}^{m_{j}}w_{ij}\right]$ and $c_{exc}E\left[\sum_{i=1}^{m_{j}}w_{ij}X_{i}\right]$), and two axonal, communication costs, (*c*_*leak*_(1 − *E*[*Z*_*j*_]) and *c*_*ap*_*E*[*Z*_*j*_]).

$$Energy\;=\;c_{leak,syn}E\left[\sum_{i=1}^{m_{j}}w_{ij}\right]\;\;+\;c_{exc}E\left[\sum_{i=1}^{m_{j}}w_{ij}X_{i}\right]\;+\;c_{leak,axo}\left(1\;-\;E\left[Z_{j}\right]\right)\;+\;c_{ap}E\left[Z_{j}\right]$$

*m*_*j*_ is the number of inputs *i* going to *j*.

*c*_*exc*_ is the scale factor of for excitatory synaptic ion-fluxes.

*c*_*leak*,*syn*_ is the scale factor of for synaptic leak cost.

*c*_*leak*,*axo*_ is the scale factor of for axonal leak cost.

*c*_*ap*_ is the scale factor of the cost of a conducted action potential.

*c*_*ap*_/*c*_*leak*_ ranges from 40:1 to 120:1 [8].

#### Error-rate

The error-rate of a neuron is calculated as the fraction of times a neuron fires to a pattern belonging to a non-preferred category. A neuron’s preferred category is defined as the category to which a neuron fires the most. As the error-rate of a neuron increases, the neuron’s specificity decreases.

### Assumptions & simplifications

Table 2 shows assumptions and simplifications of the model used here.

**Table 2:** Assumptions and simplifications.

1. Stochastic synapse formation as a function of postsynaptic firing-rate
2. Bi-directional Hebbian synaptic modification
3. Thresholded additive neurons
4. Inputs are ergodic and stationary.
5. Rate constants are appropriately matched to ergodic mixing of inputs.
6. There exist optimal firing-rates for neurons.
7. Natural selection assigns an optimal bits-per-joule.
8. The timescale of a neuron and Hebbian modification is matched to the rate of input presentations. Synaptogenesis is much slower.
9. Category-specific neurons are desirable.

### Setting ε relative to γ

**Fig 8:**
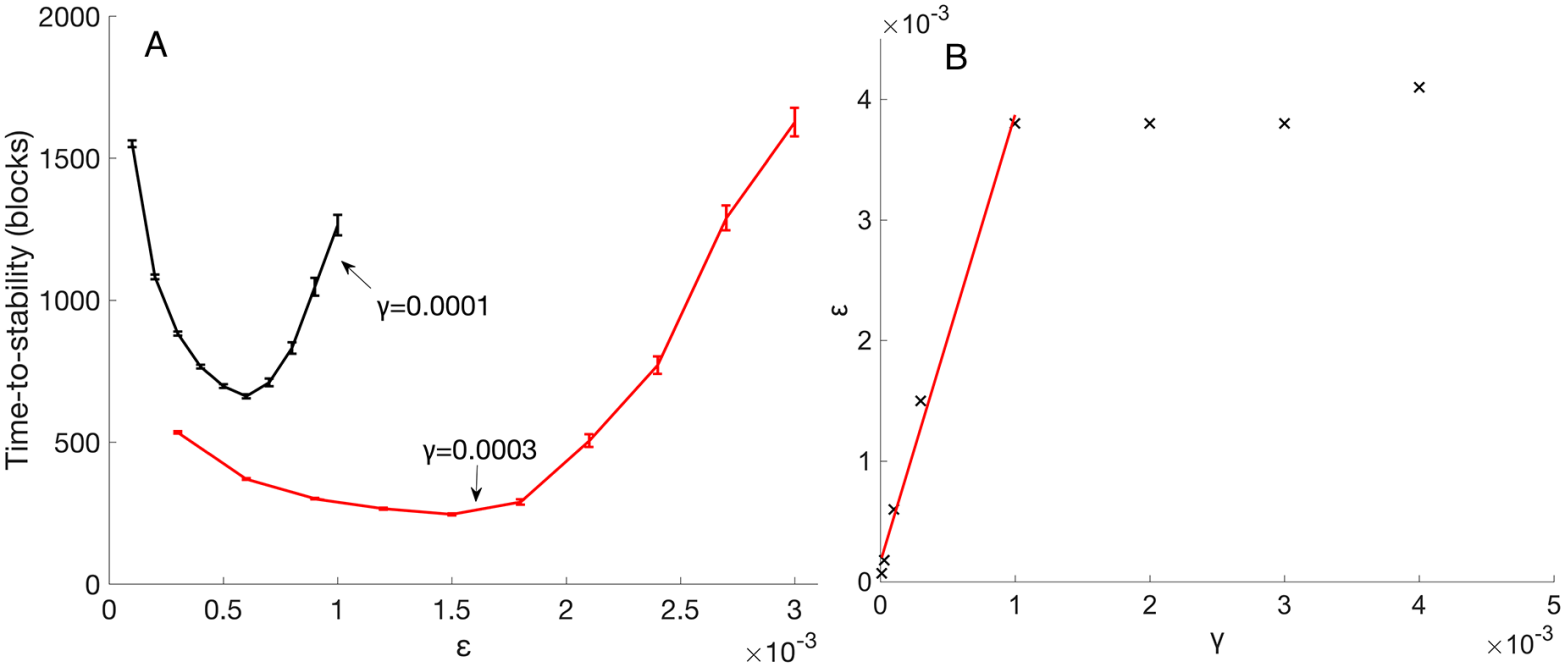
Matching ε to γ for fast development (A) Average time-to-stability is convex in ε. That is, at each value of γ, there is a value of ε that produces shortest time-to-stability. (B) Best ε for fastest development as a function of γ. For γ values larger than 0.001, the best ε is approximately the constant 0.004.

At each value of γ, there is a best value of ε that produces fast convergence to stability. For Dataset A, such ε settings were found by sweeping over a range of values and creating the empirical convex functions as seen in Fig 8A. See S1 Appendix for further remarks concerning this convexity. For γ values below 0.001 and the input sets used here, the best ε value is approximately predicted by a fixed ratio (ca. 5) between ε and γ. For γ values above 0.001, the relationship breaks down; at such γ values, an ε setting greater than 0.004 produces neurons that develop slower (Fig 8B). At even higher γ values of 0.01, neuron allocation is not proportional to category frequency (See Neuron Allocation in Results).

For Dataset C, the values of ε and γ are both 0.001.

### Input datasets

**Fig 9:**
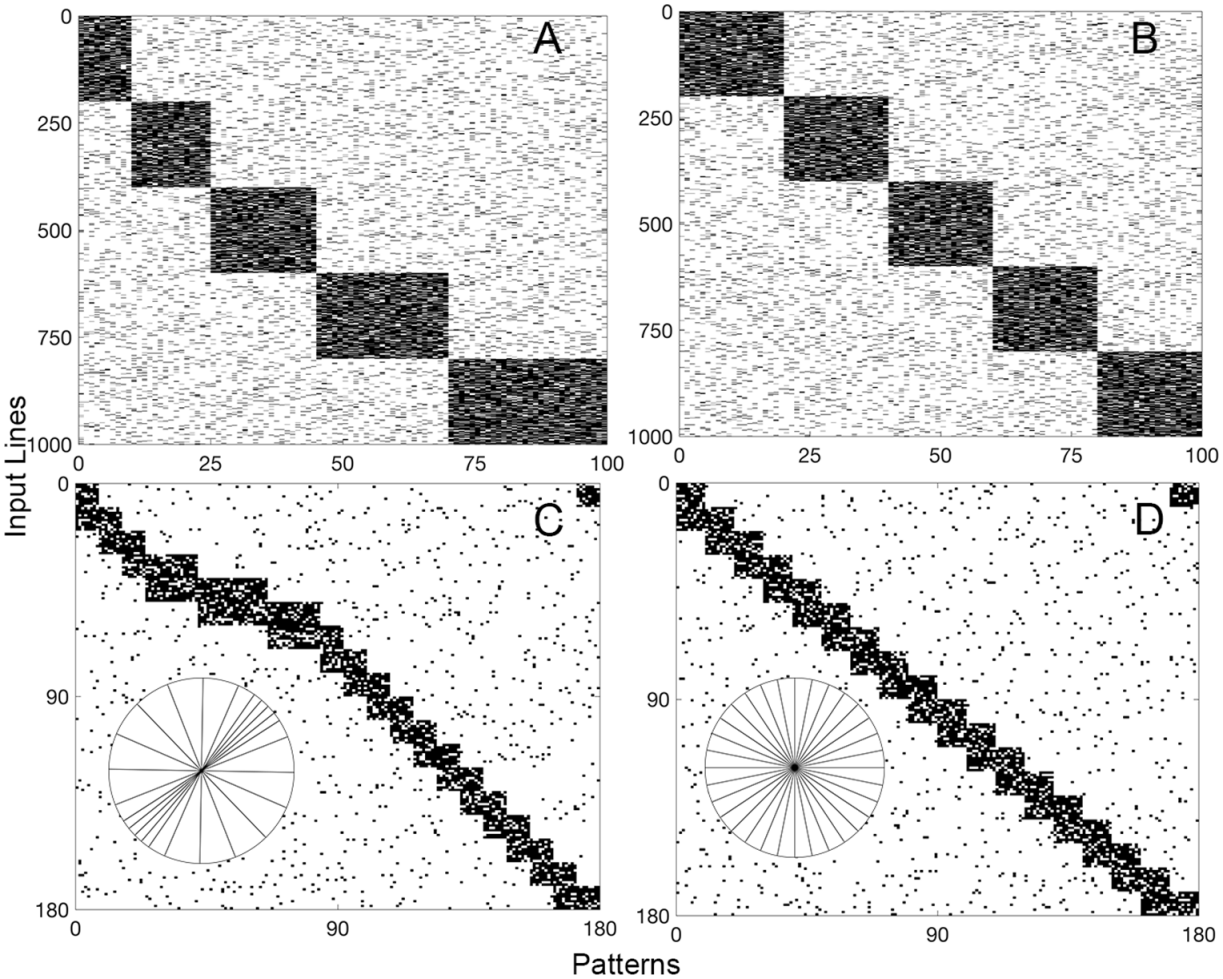
Input datasets single blocks of the datasets. Patterns are grouped together by category to aid visualization of the categories, but in simulations, the presentation order of the patterns is randomized for each block. (A) and (B) show 100-pattern blocks of Datasets A1 and A2, respectively. There are five orthogonal categories. Dataset A1 has categories with probabilities of 0.1, 0.15, 0.2, 0.25, and 0.3, and Dataset A2 has categories with the same probability of 0.2. (C) and (D) show 100-pattern blocks of the Datasets C1 and C3, respectively. Datasets C2 and C4 are not shown. The datasets mimic environments of orientation neurons in the visual system. The circular illustrations are qualitative visualizations of the orientation biases that each C dataset represents. Dataset C1 and C2 simulate experimentally manipulated environments with contours of a single orientation. Dataset C3 simulates a retinal wave-generated environment with no bias in orientation, and Dataset C4 simulates a normal visual environment with slight biases for horizontal and vertical orientations. The circular illustrations are qualitative visualizations of the datasets. See text for more details, including references to the empirical motivations.

There are two classes of input environments studied here: (A and C; Table 3, S3 Code). Datasets A1 and A2 (Fig 9A and 9B) have five orthogonal prototypes, each defined by 200 adjacent input lines valued one (i.e., “on”) and the remaining are valued zero (i.e., “off”). In Dataset A1, the prototypes have relative frequencies of 0.1, 0.15, 0.2, 0.25, and 0.3 for a total of 100 input patterns per block. Dataset A2 has equally probable categories.

**Table 3:** Dataset parameters.

Dataset	A1	A2	C1	C2	C3	C4
Input dimensions	1,000	1,000	180	180	180	180
Active lines per pattern	200	200	20	20	20	20
Number of categories	5	5	18	18	18	18
On-noise	100	manipulated	5	5	5	5
Off-noise	100	manipulated	5	5	5	5
Category probabilities	{.1, .15, .2, .25, .3}	.2 for all	.13 for 5; .1 for 4, 6; 0.04 for rest	.13 for 14; .1 for 13, 15; 0.04 for rest	.055 for all	.088 for 1, 9; .07 for 2, 8, 10, 18; .04 for rest
Block size	100	100	180	180	180	180

Random noise is added to our input environments. In Dataset A1, 100 input lines of the prototype are turned off (100 off-noise) and 100 input lines not of the prototype are turned on (100 on-noise). For each input pattern, the total number of active input lines remains constant. For Dataset A2, we manipulated the level of on- and off-noise to investigate the effects of noise in the input environment on neuronal development (See Noise-to-signal ratio constrains best ε).

The C datasets crudely mimic the input environment of orientation neurons in V1 visual cortex. Each input line represents an angle of an orientation, and thus, there is a total of 180 input lines for 180 degrees (Fig 9). (There are 180 input lines and not 360 because a line rotated 180 degrees has the same orientation.) There are 18 categories, or prototypes, each with 20 input lines. A category overlaps with its two adjacent categories. Ten of its 20 input lines are shared with one adjacent category, and the other ten of its 20 input lines are shared with the other adjacent category. The on-noise and off-noise are both 5 for the C Datasets.

The differences among datasets C1, C2, C3, and C4 are the probabilities of the orientations, or categories. The category probability is the probability that a pattern presented is derived from a given category, and it is determined by dividing the number of patterns in an input block derived from a category by the total number of patterns in an input block (See Timescales for an explanation of an input block). Such differences are used to simulate environments with various biases in orientations, where a bias for an orientation increases the probabilities of a category and its two adjacent categories. Dataset C1 and C2 mimic an experimentally manipulated, stripe-tilt environment with biases in orientations of 45 and 135 degrees, respectively (categories 5 and 14) [53]. Dataset C3 represents a retinal wave-generated environment with no bias in any orientations [54, 55]. Dataset C4 represents a normal postnatal environment with slight biases in the vertical and horizontal orientation (categories 1 and 9) [56–58].

For analysis of simulations in a more complex input environment, see S2 Appendix.

### Noise-to-signal ratio constrains best ε

**Fig 10:**
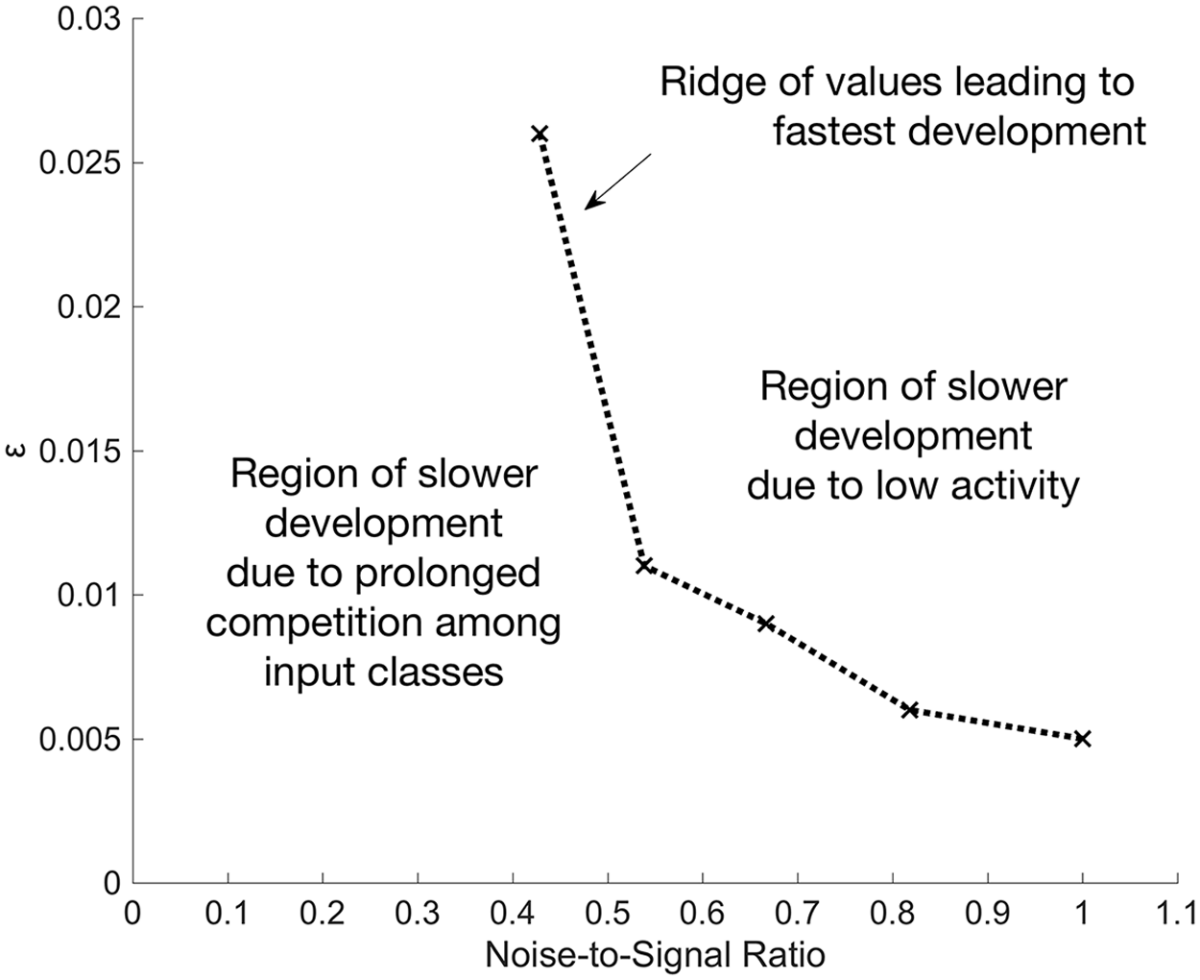
At a higher NSR, a lower ε is required for fast neuron development. A phase diagram of the shortest development time with respect to ε and NSR. On average, a neuron develops the fastest along the curve of plotted points; above or below the points, development is slower. With an ε value above the curve, a neuron develops slower because its low firing-rate is too close to the receptivity threshold, while an ε value below the curve leads to slower development because of prolonged competition among input classes (See S1 Appendix for an explanation of how slow development occurs).

At lower noise levels, larger ε values can be used to speed up development, and at higher noise levels, smaller ε values are required (Fig 10).

Input noise level is measured via the noise-to-signal ratio (NSR). The NSR of individual input vectors is calculated by dividing the number of active input lines orthogonal to the selected prototype by the number of active input lines belonging to the selected prototype. All datasets are such that the number of active input lines are the same for each pattern; i.e. the on- and off-noise are equal (See Input datasets in Methods for description of on- and off-noise).

To understand the relationship between ε and input noise, consider an input environment with no noise and an environment with an extremely high NSR. In an input environment with no noise, a neuron only needs a single synapse to maintain a high enough firing-rate, given that the probability of the corresponding input line is above ρ (receptivity threshold). With a large ε, a neuron can quickly produce just a few synapses (See S1 Appendix) and converges to stable connectivity. In contrast, a neuron in a high noise environment needs more synapses to maintain average firing-rate above ρ, and a small ε allows the neuron to amass enough synapses. When ε is small, a neuron’s synapses are also shed more slowly. Because synapses are added much faster than they can be shed, this neuron accumulates a large number of synapses.

### Time penalty for suboptimal parameter selection

Here, we show the time penalty for suboptimal parameter values as a function of input noise levels. In Fig 2, three sets of values are compared. To best understand these time penalties, consider these time penalties from Nature’s evolutionary perspective.

**Fig 11:**
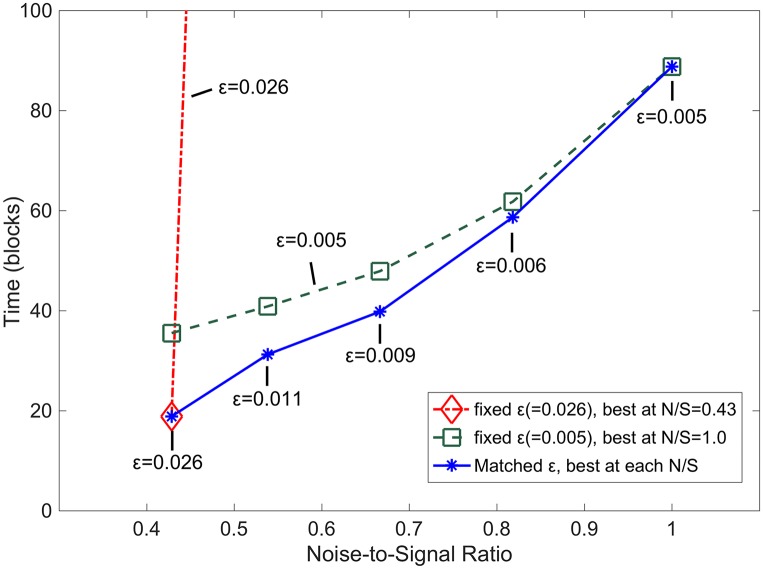
Time penalty for suboptimal parameter selection. The time penalty for suboptimal parameter selection is the difference between the dot-dashed/dashed curves and the continuous curve. This blue curve (*) is the time to stable development at each NSR using the optimal ε for each NSR. The dot-dashed and dashed curves use fixed ε values, with the dot-dashed (red) using an ε value (0.026) optimal for a low NSR (0.43) and the dashed (blue) using an ε value (0.005) optimal for a high NSR (1.0). The values of the points of the dot-dashed, red line outside of the plot are 553, 8,282, 14,790, and 14,988.

If the parameter ε could be set to suit each noise level for fastest development, then the solid blue (*) line of Fig 11 would represent the development times of neurons. The dashed and dot-dashed lines represent scenarios in which Nature does not evolve a best ε value for each noise level, but instead must chose a single value for all environments. A small ε value is best suited for a high-noise environment, and it also works well in low noise environments (shown by the dashed, green, square line). In such case, the time penalty for choosing a small ε is small for the noisy environments, but at NSR of 0.43, neurons take twice as long, on average, to develop with the single fixed ε value. This time penalty, however, is small compared to the penalties of using the large ε at all NSRs. That is, if Nature chose a large ε value best suited for a low-noise environment, the time penalty at environments with a noise level any greater than the lowest noise level, for which the ε value was chosen, would be extreme. At NSR of around 0.55, the time penalty is 520 which is around 26 times slower, and at the highest noise level of 1.0 NSR, the time penalty becomes 14,900, which is over 150 times slower than if a suitable ε value was chosen. Thus, if ε is not adjustable to specific NSRs, then Nature should choose the smaller value for robustness across all noise inputs.

## Supporting information

S1 Appendix Neuron development dynamics as a function of ε. (PDF)

S2 Appendix Results replicated with a richer input environment, dataset B. (PDF)

S3 Appendix The optimization credo. (PDF)

## References

[1] Bourgeois J-P, Jastreboff PJ, Rakic P. Synaptogenesis in visual cortex of normal and preterm monkeys: Evidence for intrinsic regulation of synaptic overproduction. Proc Natl Acad Sci U S A. 1989;86: 4297–301. 2726773 10.1073/pnas.86.11.4297 PMC287439

[2] Huttenlocher PR, Dabholkar AS. Regional differences in synaptogenesis in human cortex. J Comp Neurol. 1997;387(2):167–78. 9336221 10.1002/(sici)1096-9861(19971020)387:2<167::aid-cne1>3.0.co;2-z

[3] Elston GN, Fujita I. Pyramidal cell development: postnatal spinogenesis, dendritic growth, axon growth, and electrophysiology. Front Neuroanat. 2014;8:78 doi: 10.3389/fnana.2014.00078 25161611 10.3389/fnana.2014.00078 PMC4130200

[4] Riccomagno MM, Kolodkin AL. Sculpting neural circuits by axon and dendrite pruning. Annu Rev Cell Dev Biol. 2015;31:779–804. doi: 10.1146/annurev-cellbio-100913-013038 26436703 10.1146/annurev-cellbio-100913-013038 PMC4668927

[5] Just MA, Keller TA, Malave VL, Kana RK, Varma S. Autism as a neural systems disorder: A theory of frontal-posterior underconnectivity. Neurosci Biobehav Rev. 2012;36(4): 1292–1313. doi: 10.1016/j.neubiorev.2012.02.007 22353426 10.1016/j.neubiorev.2012.02.007 PMC3341852

[6] Keown LC, Shih P, Nair A, Peterson N, Mulvey ME, Müller R-A. Local functional overconnectivity in posterior brain regions is associated with symptom severity in autism spectrum disorders. Cell Rep. 2013;5(3): 567–72. doi: 10.1016/j.celrep.2013.10.003 24210815 10.1016/j.celrep.2013.10.003 PMC5708538

[7] Supekar K, Uddin LQ, Khouzam A, Phillips J, Gaillard WD, Kenworthy LE, Yerys BE, Vaidya CJ, Menon V. Brain hyperconnectivity in children with autism and its links to social deficits. 2013;5(3): 738–47. 10.1016/j.celrep.2013.10.001 PMC3894787 24210821

[8] Levy WB, Baxter RA. Energy efficient neural codes. Neural Comput. 1996;8(3): 531–43. 8868566 10.1162/neco.1996.8.3.531

[9] Laughlin SB, Sejnowski TJ. Communication in neuronal networks. Science. 2003;301(5641): 1870–4. doi: 10.1126/science.1089662 14512617 10.1126/science.1089662 PMC2930149

[10] Thomas BT, Blalock DW, Levy WB. Adaptive synaptogenesis constructs neural codes that benefit discrimination. PLoS Comput Biol. 2015 7 20 doi: 10.1371/journal.pcbi.1004299 26176744 10.1371/journal.pcbi.1004299 PMC4503424

[11] Low LK, Cheng HJ. Axon pruning: an essential step underlying the developmental plasticity of neuronal connections. Philos Trans R Soc Lond B Biol Sci. 2006 9 29 doi: 10.1098/rstb.2006.1883 16939973 10.1098/rstb.2006.1883 PMC1664669

[12] Paolicelli RC, Bolasco G, Pagani F, Maggi L, Scianni M, Panzanelli P, et al Synaptic pruning by microglia is necessary for normal brain development. Science. 2011;333(6048): 1456–8. doi: 10.1126/science.1202529 21778362 10.1126/science.1202529

[13] Chechik G, Meilijson I, Ruppin E. Synaptic pruning in development: a computational account. Neural Comput. 1998;10(7): 1759–77. 9744896 10.1162/089976698300017124

[14] Purves D, Lichtman JW. Elimination of synapses in the developing nervous system. Science. 1980;210: 135–7. 10.1126/science.7414326 7414326

[15] Van Ooyen A, van Pelt J. Activity-dependent outgrowth of neurons and overshoot phenomena in developing neural networks. J Theor Biol. 1994;167: 27–43.

[16] Levy WB, Desmond NL. The rules of elemental synaptic plasticity In: Levy WB, Anderson JA, Lehmkuhle S, editors. Synaptic Modification, Neuron Selectivity, and Nervous System Organization. Hillsdale, NJ: Lawrence Erlbaum Associates; 1985 P. 105–121.

[17] Levy WB. Contrasting rules for synaptogenesis, modification of existing synapses, and synaptic removal as a function of neuronal computation. Neurocomputing. 2004;58–60: 343–350.

[18] Bienenstock EL, Cooper LN, Munro PW. Theory for the development of neuron selectivity: orientation specificity and binocular interaction in visual cortex. J Neurosci. 1982;2(1): 32–48. 7054394 10.1523/JNEUROSCI.02-01-00032.1982 PMC6564292

[19] Iglesias J, Eriksson J, Grize F, Tomassini M, Villa AEP. Dynamics of pruning in simulated large-scale spiking neural networks. Biosystems. 2005;79(1–3): 11–20. doi: 10.1016/j.biosystems.2004.09.016 15649585 10.1016/j.biosystems.2004.09.016

[20] Butz M, Ooyen AV. A simple rule for dendritic spine and axonal bouton formation can account for cortical reorganization after focal retinal lesions. PLoS Comput Biol. 2013 10 23 doi: 10.1371/journal.pcbi.1003259 24130472 10.1371/journal.pcbi.1003259 PMC3794906

[21] Fauth M, Wörgötter F, Tetzlaff C. The formation of multi-synaptic connections by the interaction of synaptic and structural plasticity and their functional consequences. PLoS Comput Biol. 2015 1 15 doi: 10.1371/journal.pcbi.1004031 25590330 10.1371/journal.pcbi.1004031 PMC4295841

[22] Levy WB, Colbert CM, Desmond NL. Elemental adaptive processes of neurons and synapses: a statistical/computational perspective In: Gluck MA, Rumelhart DE, editors. Neuroscience and Connectionist Theory. Hillsdale, NJ: Lawrence Erlbaum Associates; 1990 p. 187–235.

[23] Adelsberger-Mangan DM, Levy WB. Information maintenance and statistical dependence reduction in simple neural networks. Biol Cybern. 1992;67: 469–477. 1391119 10.1007/BF00200991

[24] Adelsberger-Mangan DM, Levy WB. Adaptive synaptogenesis constructs networks that maintain information and reduce statistical dependence. Biol Cybern. 1993;70: 81–87. 8312400 10.1007/BF00202569

[25] Adelsberger-Mangan DM, Levy WB. The influence of limited presynaptic growth and synapse removal on adaptive synaptogenesis. Biol Cybern. 1994;71: 461–68. 7993933 10.1007/BF00198922

[26] Adelsberger-Mangan DM, Levy WB. Adaptive synaptogenesis constructs networks which allocate network resources by category frequency. IEEE Trans Neural Netw. 1994 doi: 10.1109/icnn.1994.374566

[27] Colbert CM, Fall CP, Levy WB. Using adaptive synaptogenesis to model the development of ocular dominance in kitten visual cortex In: Eeckman FH, editors. Computation in Neurons and Neural Systems. Norwell, MA: Kluwer Academic Publishers; 1994 p. 139–144.

[28] Wiesel TN, Hubel DH. Effects of visual deprivation on morphology and physiology of cells in the cat’s lateral geniculate body. J Neurophysiol. 1963;26: 978–93. 14084170 10.1152/jn.1963.26.6.978

[29] Levy WB, Baxter RA. Energy-efficient neuronal computation via quantal synaptic failures. J Neurosci. 2002;22(11): 4756–5. 12040082 10.1523/JNEUROSCI.22-11-04746.2002 PMC6758790

[30] Berger T, Levy WB. A mathematical theory of energy efficient neural computation and communication. IEEE Trans Inf Theory. 2010; 56: 852–74.

[31] Levy WB, Ju H, Baxter RA, Colbert CM. Controlling information flow and energy use via adaptive synaptogenesis. 2016 Annual Conference on Information Science and Systems (CISS).

[32] Balasubramanian V, Kimber D, Berry MJ. Metabolically efficient information processing. Neural Comput. 2001;13(4): 799–815. 11255570 10.1162/089976601300014358

[33] Hamilton W. The moulding of senescence by natural selection. Journal of Theoretical Biology. 1966;12(1): 12–45. 6015424 10.1016/0022-5193(66)90184-6

[34] Trivers RL. Parent-offspring conflict. Am Zool. 1974;14(1): 249–64.

[35] Dawkins R. The selfish gene. 1st ed Oxford: Oxford University Press; 1976.

[36] Stearns SC. The evolution of life histories. New York: Oxford University Press; 1992 pp. 161.

[37] Charlesworth B. Evolution in age-structured populations. 2nd edn Cambridge: University Press; 1994 pp. 217–23.

[38] Roff D. Life history evolution. Sunderland, MA: Sinauer Associates, Inc; 2002 pp. 230–6.

[39] Parker GA, Maynard Smith J. Optimality theory in evolutionary biology. Nature. 1990;348: 27–33.

[40] Missler M, Wolff A, Merker H, Wolff JR. Pre- and postnatal development of the primary visual cortex of the common marmoset. II. Formation, remodelling, and elimination of synapses as overlapping processes. J Comp Neurol. 1993 7 1 doi: 10.1002/cne.903330105 8340496 10.1002/cne.903330105

[41] Bourgeois JP, Rakic P. Changes in synaptic density in the primary visual cortex of the macaque monkey from fetal to adult stage. J Neurosci. 1993;13(7): 2801–20. 8331373 10.1523/JNEUROSCI.13-07-02801.1993 PMC6576672

[42] Innocenti GM. Exuberant development of connections, and its possible permissive role in cortical evolution. Trends Neurosci. 1995;18(9): 397–402. 7482805 10.1016/0166-2236(95)93936-r

[43] Zehr JL, Todd BJ, Schulz KM, McCarthy MM, Sisk CL. Dendritic pruning of the medial amygdala during pubertal development of the male Syrian hamster. Dev Neurobiol. 2006 3 22 doi: 10.1002/neu.20251 16555234 10.1002/neu.20251

[44] Drzewiecki CM, Willing J, Juraska JM. Synaptic number changes in the medial prefrontal cortex across adolescence in male and female rats: A role for pubertal onset. Synapse. 2016 6 10 doi: 10.1002/syn.21909 27103097 10.1002/syn.21909 PMC4945496

[45] Huttenlocher PR. Synaptic density in human frontal cortex—developmental changes and effects of aging. Brain Res. 1979;163(2): 195–205. 427544 10.1016/0006-8993(79)90349-4

[46] Huttenlocher PR, de Courten C, Garey LJ, Van der Loos H. Synaptogenesis in human visual cortex—evidence for synapse elimination during normal development. Neurosci Lett. 1982;33(3): 247–52. 7162689 10.1016/0304-3940(82)90379-2

[47] Huttenlocher PR, de Courten C. The development of synapses in striate cortex of man. Hum Neurobiol. 1987;6(1): 1–9. 3583840

[48] Zecevic N, Rakic P. Synaptogenesis in monkey somatosensory cortex. Cereb Cortex. 1991;1(6): 510–23. 1822755 10.1093/cercor/1.6.510

[49] Petanjek Z, Judaš M, Šimic G, Rasin MR, Uylings HB, Rakic P, Kostovic I. Extraordinary neoteny of synaptic spines in the human prefrontal cortex. Proc Natl Acad Sci U S A. 2011;108(32): 13281–6. doi: 10.1073/pnas.1105108108 21788513 10.1073/pnas.1105108108 PMC3156171

[50] Koss WA, Belden CE, Hristov AD, Juraska JM. Dendritic remodeling in the adolescent medial prefrontal cortex and the basolateral amygdala of male and female rats. Synapse. 2013 10 15 doi: 10.1002/syn.21716 24105875 10.1002/syn.21716

[51] Butz M, Lehmann K, Dammasch IE, Teuchert-Noodt G. A theoretical network model to analyse neurogenesis and synaptogenesis in the dentate gyrus. Neural Netw. 2006;19: 1490–505. doi: 10.1016/j.neunet.2006.07.007 17014989 10.1016/j.neunet.2006.07.007

[52] McCulloch WS, Pitts W. A logical calculus of the ideas immanent in nervous activity. Bull Math Biophys. 1943;5: 115–33. 2185863

[53] Blasdel GG, Mitchell DE, Muir DW, Pettigrew JD. A physiological and behavioural study in cats of the effect of early visual experience with contours of a single orientation. J Physiol. 1977;265(3): 615–36. 853380 10.1113/jphysiol.1977.sp011734 PMC1307838

[54] Shatz CJ. Emergence of order in visual system development. Proc Natl Acad Sci U S A. 1996;93(2): 602–8. 8570602 10.1073/pnas.93.2.602 PMC40098

[55] Feller MB, Butts DA, Aaron HL, Rokhsar DS, Shatz CJ. Dynamic processes shape spatiotemporal properties of retinal waves. 1997;19(2): 293–306. 10.1016/s0896-6273(00)80940-x 9292720

[56] Switkes E, Mayer MJ, Sloan JA. Spatial frequency analysis of the visual environment: Anisotropy and the carpentered environment hypothesis. Vision Res. 1978;18(10): 1393–9. 726283 10.1016/0042-6989(78)90232-8

[57] Hansen BC, Essock EA. A horizontal bias in human visual processing of orientation and its correspondence to the structural components of natural scenes. J Vis. 2004;4(5): 5. 10.1167/4.12.5 15669910

[58] Torralba A, Oliva A. Statistics of natural image categories. Network. 2003;14: 391–412. 12938764

