# Supplementary material for: Limited synapse overproduction can speed development but sometimes with long-term energy and discrimination penalties
Source: PLoS Comput Biol. 2017 Sep 22;13(9):e1005750. doi: 10.1371/journal.pcbi.1005750 (PMC5627944; doi:10.1371/journal.pcbi.1005750)
Supplement: S1 Appendix — (PDF) [file pcbi.1005750.s001.pdf]

S1 Appendix: Neuron development dynamics as a function of ε

Varying ε, given a fixed γ value, produces different development times. For any dataset and any γ, there is an optimal ε value in the sense that this ε produces fastest development while achieving, ca., the desired firing-rate. That is, raising or lowering values of ε around this optimal value prolongs development (Fig 8, S1 Fig 3).

To understand the non-monotonic relationship between a neuron's ε and development time, this appendix examines the developmental dynamics of representative neurons evolved with large (0.003), intermediate (0.0015), or low (0.0003) values of ε, given a fixed value of γ (=0.0003); by design, the intermediate value produces the fastest convergence to stable connectivity.

The slow development of a neuron with a larger ε value arises from high variations of firing-rate when firing-rate is close to the receptivity threshold ρ (Recall that when the firing-rate is above ρ, synaptogenesis is turned off. In turn, this high activity variation is due to a mismatch of ε, block size, and mixing rates.) Its slow development is representative of the 1,000 large-ε neurons simulated in this study. At block 200, the neuron seems to have converged to stable connectivity. However, at around block 600 (indicated by the first arrow), the firing-rate (red) plunges below ρ. Although firing-rate continually undergoes oscillations due to noisy perturbations of the input patterns, by chance, the firing-rate drops by a substantial amount further at block 600, going below ρ; thus, at this time, synaptogenesis occurs, and synapse number (black line) transiently jumps upward. This phenomenon occurs again at around block 2100, indicated by the second arrow. Since a simulation is designed to end 200 blocks after the last occurrence of synaptogenesis or shedding, these transient jumps in synapse number are the cause of the delayed convergence to stable connectivity.

The slow development of a neuron with a small ε value is brought about by two factors. The first is a large accumulation of synapses during development. Intuitively, when ε is small, a neuron's weights take longer to converge, and thus, synapses are also shed more slowly. Because synapses are added much faster

than they can be shed, such a neuron accumulates a large number of synapses (67 at maximum synapse number versus the maximum synapse number of 30 for a neuron governed by an intermediate ε value).

Such buildup of synapses leads to the second factor, i.e., with so many input connections, a neuron is pulled in the directions of several categories, a competition that slows a neuron's convergence to a single category. Fig 2 illustrates this effect by plotting the cosine between the neuron's time-evolving, synaptic weight vector and the vector of each category prototype. That is, the larger the cosine value, the more closely the neuron's weights are aligned to the prototype of a category. In S1 Fig 2A, a neuron develops governed by an intermediate ε. Note that it begins to align with the prototype of category 4 a little before block 100. On the other hand, in S1 Fig 2B, a neuron develops governed by a small ε. First, this neuron does not converge until a little before block 500. Second, as illustrated by the interweaving of the five different cosines, the neuron is being pulled in five different direction before it starts heading toward one and away from the other four.

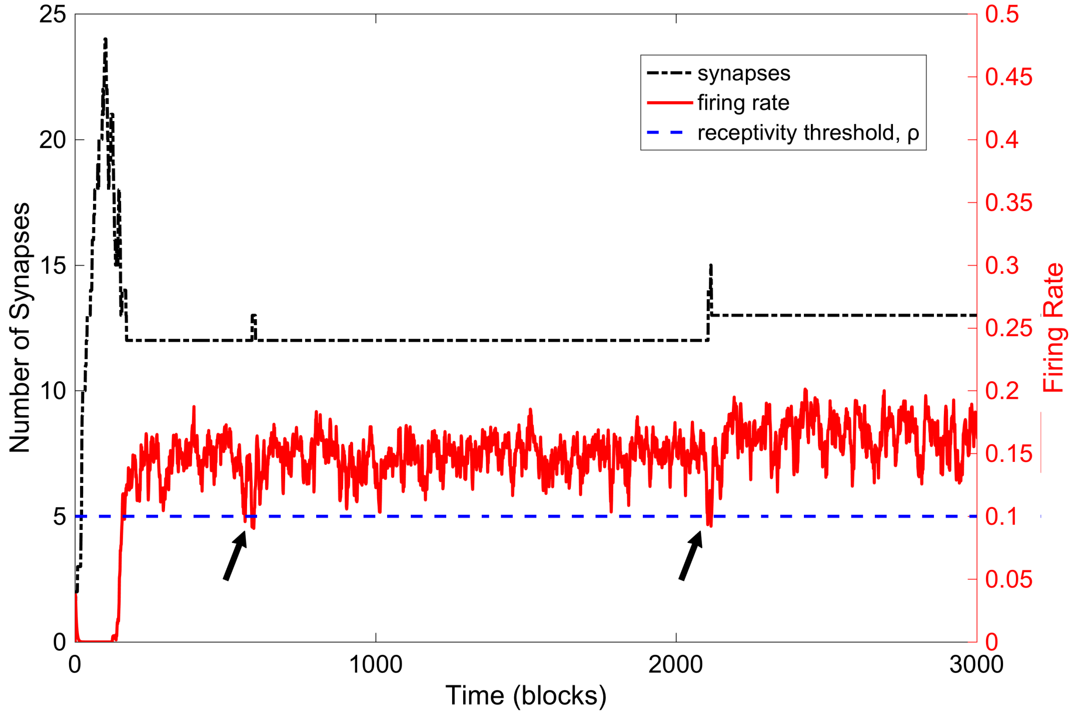

S1 Fig 1. Relatively large ε values prolong time to stabilize synapse number

The synapse count and firing-rate of a representative neuron during development. Whenever the activity drops below the receptivity threshold (noted by arrows), the neuron undergoes synaptogenesis of multiple synapses in a few cycles, resulting in slower convergence. The values of ε and γ are 0.003 and 0.0003, respectively, and the neuron is the first of the 1,000 simulated with these values.

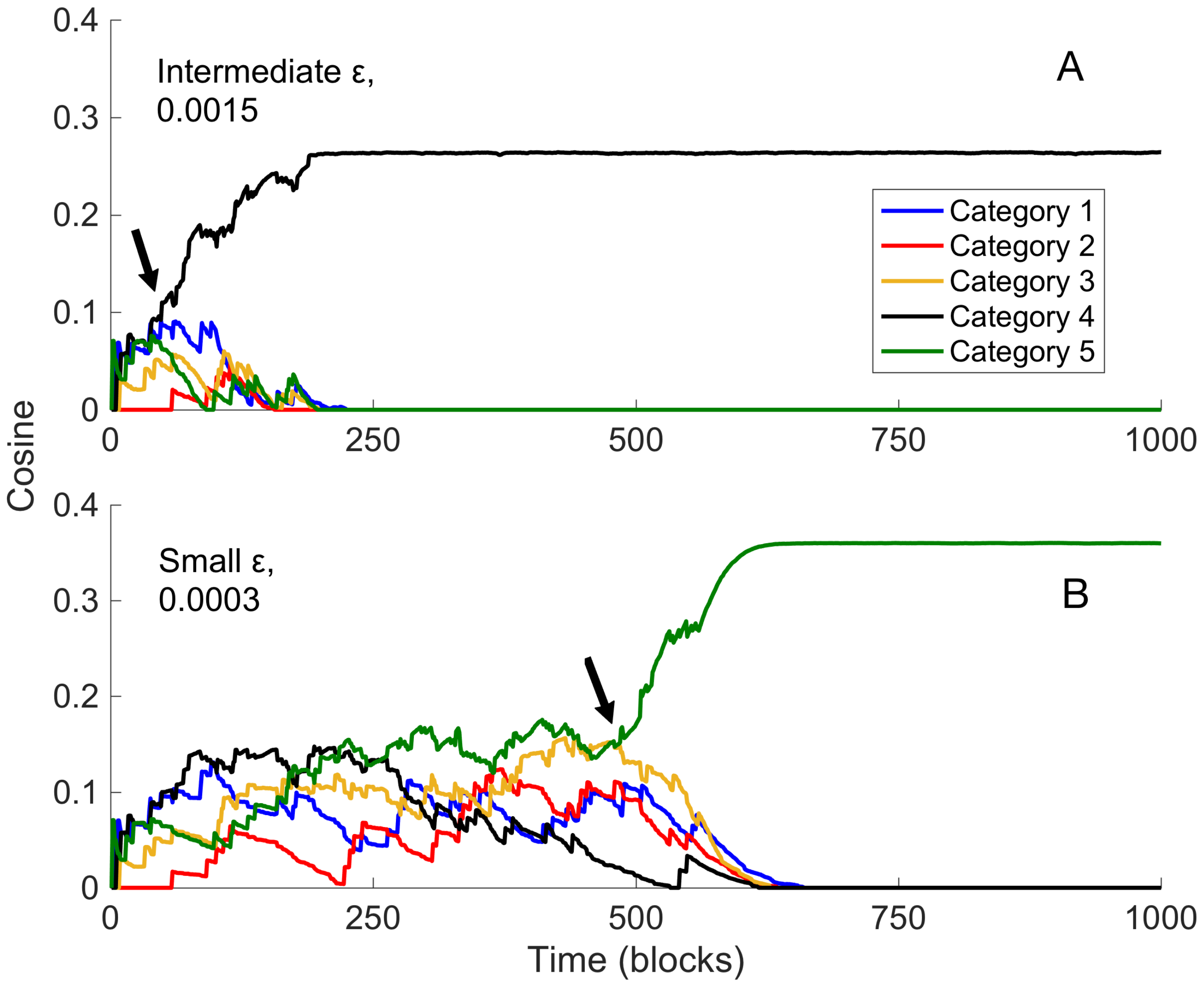

S1 Fig 2. Relatively small ε values prolong competition among categories

Smaller ε values allow more categories to compete for control of a neuron. Thus, small ε prolongs development in this indirect manner in addition to slowing the convergence of weights via synaptic weight modification. The arrows in A and B indicate the block at which one category starts to dominate the neuron's internal excitation. While category 4 in A becomes dominant at less than 100 blocks (arrow), category 5 in B becomes dominant at around 500 blocks (arrow). Each line is the cosine similarity between the weights of a neuron and the prototype of category. Higher

cosines indicate greater alignment between the neuron's weights and a category vector's prototype. These two neurons come from Dataset A1 (a 1,000-dimension input space) simulations. The value of γ is 0.0003, and the two simulations are representative.

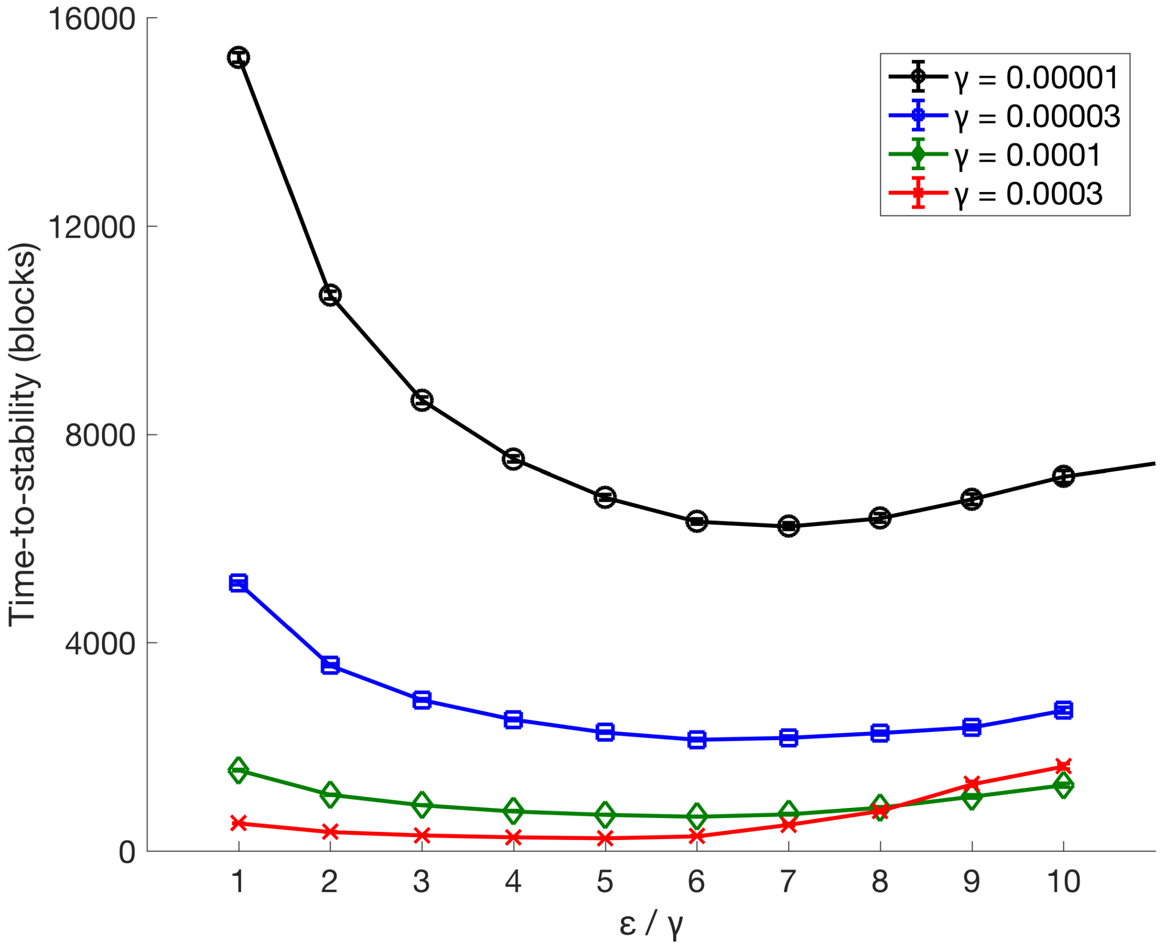

S1 Fig 3. Matching ε to γ for fast development.

Average time-to-stability is convex in ε/γ. That is, at each value of γ, there is a value of ε that produces shortest time-to-stability, and the value of the ratio ε/γ that produces shortest time-to-stability is consistent over a large range of γ. The convexity of the curves for γ at 0.001 and 0.003 are more obvious in Fig 8 of the main text.
