# Supplementary material for: Limited synapse overproduction can speed development but sometimes with long-term energy and discrimination penalties
Source: PLoS Comput Biol. 2017 Sep 22;13(9):e1005750. doi: 10.1371/journal.pcbi.1005750 (PMC5627944; doi:10.1371/journal.pcbi.1005750)
Supplement: S2 Appendix — (PDF) [file pcbi.1005750.s002.pdf]

S2 Appendix

Using dataset B, a richer input environment, replicates the findings that use dataset A (S1 Fig 1). In particular, S2 Fig 2, S2 Fig 3, and S2 Fig 4 correspond to Fig 3, Fig 4, and Fig 5 that use dataset A in the main manuscript. A fixed value of ε of 0.01 was used for all simulations here. In these simulations, the algorithm has been updated such that the three parts of the algorithm—synaptic modification, shedding, and synaptogenesis—all occur in each time step.

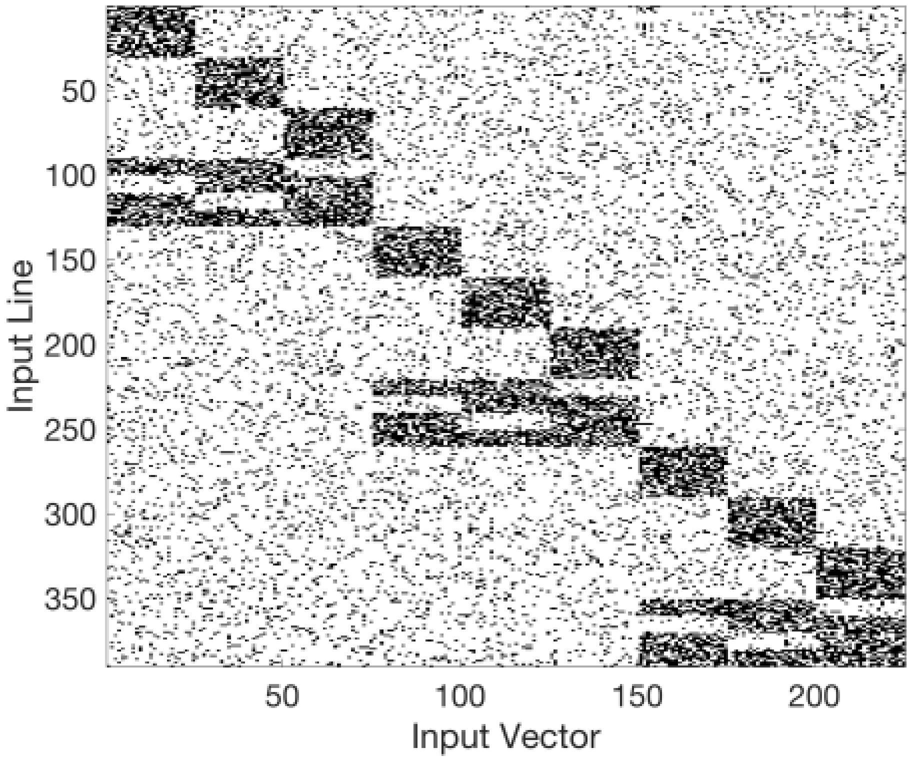

S2 Fig 1. Dataset B.
A 225-pattern block of Dataset B. Its nine categories are grouped into three orthogonal super-categories, in which a subcategory within a super-category has input lines shared by the two other categories as well as those that are not shared. Each category has a probability of 0.11.

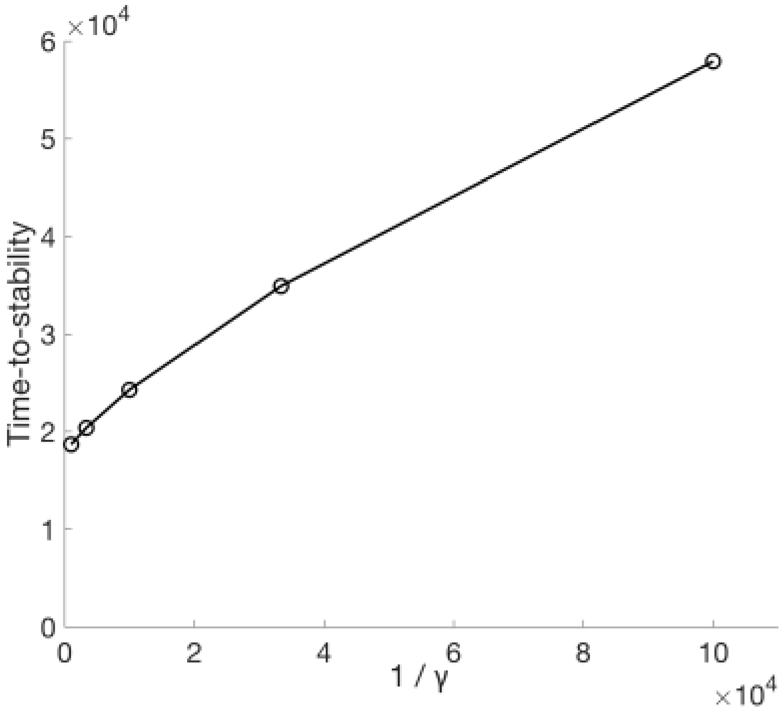

S2 Fig 2. Time-to-stability is a linearly related to the inverse of the probability of synapse formation. Increasing γ speeds development, and time-to-stabilize is proportional to the inverse of γ. The relation spans a considerable range of values of γ: 0.0001, 0.0003, 0.001, 0.003, and 0.01. These simulations use fixed values of ε at 0.01. Each datapoint is a simulation of 40 postsynaptic neurons.

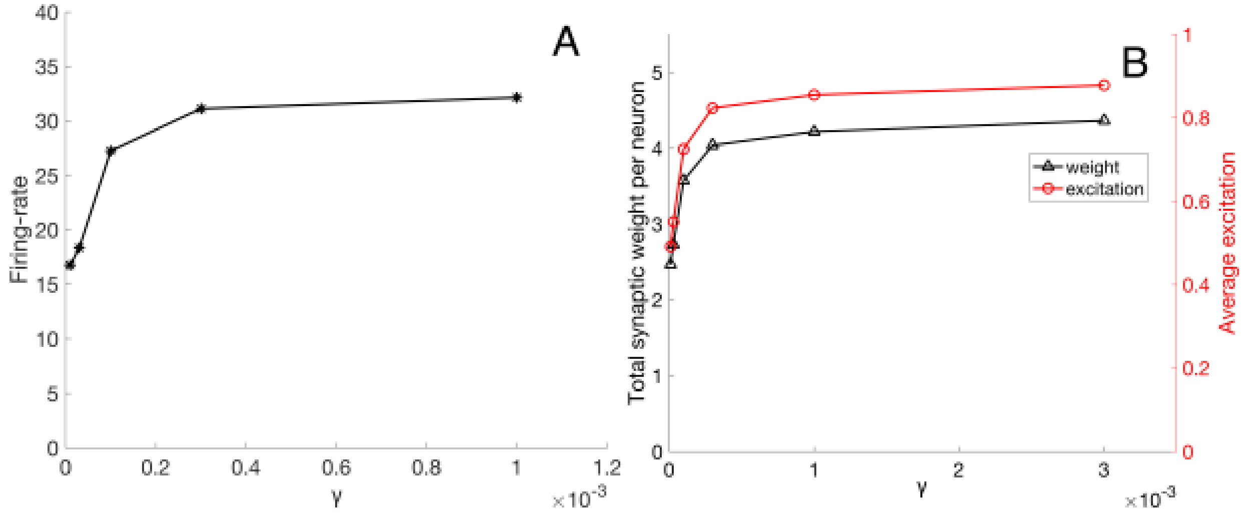

S2 Fig 3. Increasing γ produces energetically costlier neurons

At stability, neurons that develop under larger γ's use more energy in these ways. (A) Larger γ produces neurons with greater firing-rates. Communication costs rise with more action potentials because an action potential's energy-cost is about 100 times the leak energy-costs over the same time interval. (B) Larger γ produces neurons with greater total synaptic weight and greater average excitation. The computational costs arise from maintenance of synapses, proportional to the total synaptic weight, and the average use of the synapses, proportional to average synaptic activation. Each datapoint is a simulation of 40 postsynaptic neurons. Without knowing the constants c_{ap}, $c_{leak,axo}$, c_{exc}, and $c_{leak,syn}$, the y-axes do not afford comparisons across dependent variables.

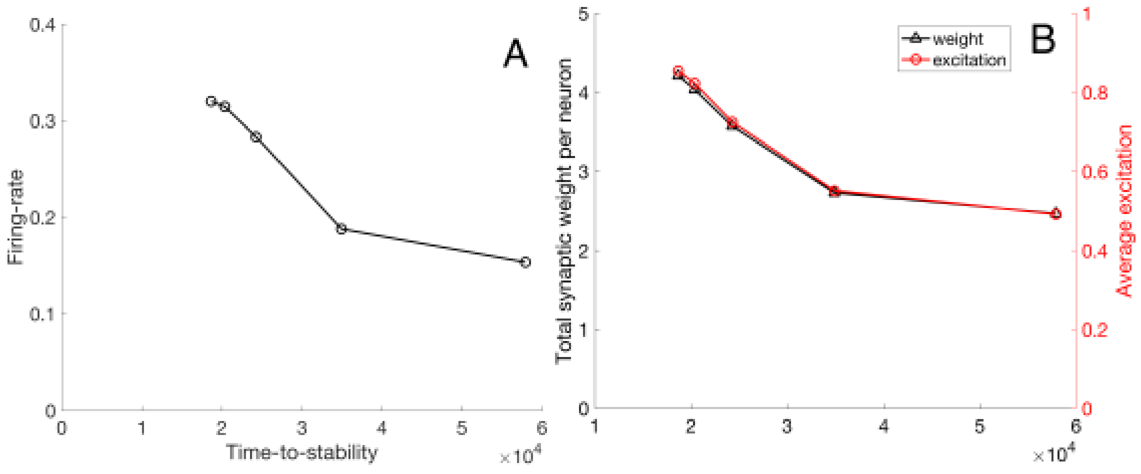

S2 Fig 3. Faster development correlates with more energetically expensive neurons.
(A) Neurons with shorter time-to-stability have more frequent action potentials and thus greater communication costs on average. (B) Neurons with shorter time-to-stability have greater total synaptic weights and greater average synaptic activation. The values of γ for the points from right to left are 0.0001, 0.0003, 0.001, 0.003, and 0.01. These simulations use fixed values of ε at 0.01. Each datapoint is a simulation of 40 neurons.
