# Supplementary material for: Limited synapse overproduction can speed development but sometimes with long-term energy and discrimination penalties
Source: PLoS Comput Biol. 2017 Sep 22;13(9):e1005750. doi: 10.1371/journal.pcbi.1005750 (PMC5627944; doi:10.1371/journal.pcbi.1005750)
Supplement: S3 Appendix — (PDF) [file pcbi.1005750.s003.pdf]

S3 Appendix: The optimization credo

The optimization approach for understanding evolved function in the context of neural computation and neuronal communication is not new (e.g., Levy and Baxter 1996, 2002; Laughlin and Sejnowski 2003). Its justification hinges on the idea that given enough time and given enough stability of the local environment (e.g., inside the organism or in a brain region), then evolution through natural selection (i.e., Nature), will have created an optimal construction satisfying a particular set of constraints. Under such assumptions, a way to understand biological function — from Nature's perspective — is to construct, parameterize, and validate one or more optimization hypotheses. As for any hypothesis (quantitative or otherwise), the goal of empirical research is to quantify, disprove, or modify the hypothesis via relevant biological observations.

The second sentence of the last previous paragraph requires some comments. Whatever the objective function being considered (e.g., raising fertile offspring, maintaining electrolyte balance, or perceiving and manipulating objects in the world), the existence of the same solution in living species that have diverged for tens if not hundreds of millions of years, is enough to support the argument that Nature has found some kind of optimal solution. To be a little more specific, consider the relative constancy of the general plan of the mammalian forebrain (its regions and subregions) and, as well, the conservation of neuron types and transmitter actions across species.

The third sentence of the first paragraph also requires a small comment. Certain problems faced by organisms have a particularly direct effect on the fundamental quantity of evolution, called fitness or as it must sometimes be extended, inclusive fitness. That is, one considers phenotypes that are so obviously important to fitness that one should assume such phenotypes are strongly controlled by natural selection. Here, we *assume* three such phenotypes including time-to-develop, the quality of neural computations, and the energetic cost of neural computation and communication. Moreover, when, for example, a pair of phenotypes are in conflict, then one has reason to inquire what are the predicted characteristics of the

evolved compromise. Such a theory of optimal compromises informs the empirical scientist what to measure, across species or across brain regions, in an effort to understand Nature in her terms, i. e., quantify the compromise in different situations to evaluate the goodness of theory. Here, the proposed phenotypes that are at odds with each other are time to develop (a direct cost to the parent(s)) vs. adult performance (a direct cost to the offspring).

Finally, we note that in the scheme of biological sciences, neuroscience is a late-comer to the optimization approach for understanding biological functions; see for example (Maynard Smith 1978; Oster and Wilson 1978; Alexander 1982; Krebs and McCleery 1984; Stephens and Krebs 1986).

References

1. Levy WB, Baxter RA. Energy efficient neural codes. Neural Comput. 1996;8(3): 531-43.
2. Levy WB, Baxter RA. Energy-efficient neuronal computation via quantal synaptic failures. J Neurosci. 2002;22(11): 4756-5.
3. Laughlin SB, Sejnowski TJ. Communication in neuronal networks. Science. 2003;301(5641): 1870-4.
4. Maynard Smith J. Optimization theory in evolution. Annu Rev Ecol Syst. 1978;9:31-56.
5. Oster GF, Wilson EO. Caste and ecology in the social insects. New Jersey: Princeton University Press; 1978.
6. Alexander RM. Optima for animals. London: Edward Arnold; 1982.
7. Krebs JR, McCleery RH. Optimization in behavioural ecology. In: Krebs JR, Davies NB, editors. Behavioural ecology, an evolutionary approach. 2nd ed. Oxford: Blackwell; 1984. pp. 91-121.
8. Stephens DW, Krebs JR. Foraging theory. New Jersey: Princeton University Press; 1986.
